# Natterin bridges IFN-φ1 and non-canonical inflammasome pathways via *CRFB1*/Gbp4 to license Caspy2-mediated antibacterial immunity

**DOI:** 10.3389/fcimb.2025.1686758

**Published:** 2025-10-27

**Authors:** Darlan Gusso, Felipe Justiniano Pinto, Aline Ingrid Andrade-Barros, Jefferson Thiago Gonçalves Bernardo, Carlos DeOcesano-Pereira, Monica Lopes-Ferreira, Carla Lima

**Affiliations:** ^1^ Plataforma Zebrafish of the Laboratory of Applied Toxinology (CeTICs/FAPESP), Butantan Institute, São Paulo, Brazil; ^2^ Centre of Excellence in New Target Discovery (CENTD), Butantan Institute, São Paulo, Brazil

**Keywords:** Natterin, IFN-I, Caspy2, Gbp4/GSDME, inflammasome, *salmonella typhimurium*, transcriptional regulation, host defense

## Abstract

The Natterin protein family represents an evolutionarily conserved group of immune effectors in teleosts, yet its broader regulatory role in host defense remains poorly understood. Here, we demonstrate that Natterin functions as a master upstream regulator, orchestrating a critical immune network that integrates type I interferon (IFN-I) signaling with non-canonical inflammasome activation during *Salmonella Typhimurium* (ST) challenge. Using wild-type embryos treated with IFN-I neutralizing antibody followed by the use of natterin (loc795232) knockout (KO) embryos generated by CRISPR/Cas9 and integrated approaches—including RT-qPCR, Western blotting, immunohistochemistry, and behavioral assays—we found that its absence completely abrogates the ST-induced IFN-I response, including the ablation of the interferon regulatory factors *irf3* and *irf7* and the IFN-φ1 receptor *crfb1*. Consequently, Natterin deficiency prevented the expression of the LPS sensor GBP4 and the proteolytic maturation of the inflammatory caspases Caspy and Caspy2. This disruption abolished downstream *gsdme-a/b* expression, which may result in the non-formation of pores. The critical role of IFN-I signaling was independently confirmed by its neutralization in wild-type embryos, which abolished the protein-level localization of IL-1β and IFN-β and mirrored the KO phenotype. Functionally, this disruption led to a sixfold increase in mortality and exacerbated ST-induced pathogenesis. Our results establish Natterin not merely as an effector molecule but as a pivotal regulator that integrates IFN-I and inflammasome signaling, orchestrating a coordinated immune response essential for host survival. This work reveals a previously unrecognized level of regulation in teleost innate immunity with significant evolutionary parallels to mammalian defense mechanisms.

## Graphical Abstract

Proposed mechanism for Natterin-dependent inflammasome activation in zebrafish defense against *Salmonella Typhimurium* (ST). Natterin is essential for coordinating a protective immune response against ST by bridging type I interferon (IFN-I) signaling to inflammasome activation. It regulates the *IRF3*-7/*crfb1* axis to drive the production and binding of IFNφ1, respectively. This IFN-I response is a critical prerequisite, as it enables the assembly of a Caspy-Caspy2/GBP4 inflammasome complex for cytosolic LPS sensing and subsequent maturation of IL-1β, gasdermin-E activation, pore formation and the release of IL-1β. GSDME pores also facilitate K^+^ efflux. The essential role of this pathway is underscored by the finding that neutralization of IFN-I phenocopies the immune impairment observed in natterin-knockout embryos, abolishing inflammasome activation and IL-1β–mediated immunity.

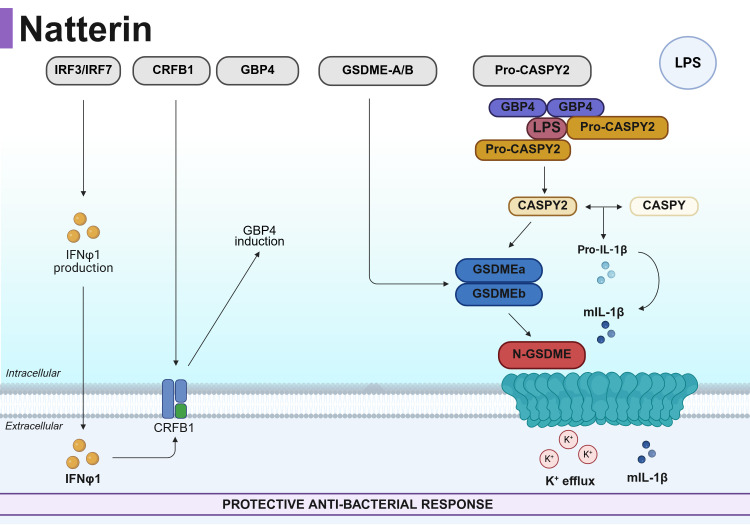

## 1 Introduction

Comprising over 30,000 species, teleost fishes represent nearly half of all extant vertebrates, a remarkable diversity rooted in their complex genomic history. This evolutionary success was driven by ancient whole-genome duplication events followed by lineage-specific gene loss (Dehal and Boore, 2005; Smith and Keinath, 2015) and the teleost-specific genome duplication (TGD), which collectively expanded their genetic repertoire and facilitated vast morphological and ecological adaptations (Taylor et al., 2003; Steinke et al., 2006). Importantly, these genomic events also contributed to the sophistication of the teleost innate immune system, including the emergence of specialized immune effector molecules such as the Natterin family.

Despite this expanded toolkit, the specific mechanisms bridging different immune pathways in teleosts remain poorly understood, particularly the crosstalk between inflammatory and interferon-based defenses. This knowledge gap motivates the present study.

Zebrafish (Danio rerio) has emerged as a powerful model for deciphering conserved yet diversified immune mechanisms. Its genome encodes a broad array of pattern recognition receptors, including Toll-like receptors (TLRs), C-type lectins (CLRs), and multiple NOD-like receptor (NLR) families, alongside key components of the interferon (IFN) pathway, such as the highly conserved STING, *IRF3*, and *IRF7* (Altmann et al., 2003; Howe et al., 2013; Guan et al., 2024; Ghani et al., 2024). Notably, zebrafish also possess lineage-specific adaptations like Caspy2, which regulates non-canonical inflammasome activation (Tyrkalska et al., 2016; Yang et al., 2018).

Among the unique immune effectors conserved from fish to invertebrates is the Natterin family (Lima et al., 2021a)—pore-forming proteins that provide protection against pathogens and environmental stressors of several aquatic species such as the Atlantic salmon Salmo salar (Jodaa Holm et al., 2016), the lamprey Lampetra morii (Xue et al., 2012; Wu et al., 2017); the Atlantic cod Gadus morhua (Rajan et al., 2017); the common carp Cyprinus carpio (Neave et al., 2017); the zebra mussel Dreissena polymorpha (Leprêtre et al., 2020) and the Arctic char Cyclopterus lumpus (Patel and Brinchmann, 2017; Patel et al., 2019), and adult zebrafish (Chen et al., 2018); or that induce inflammation in a heterologous mouse models (Lima et al., 2021b, 2022). In addition to fish, Natterin-like proteins were also identified as important components in the innate immune response of the plant Arabidopsis thaliana after bacterial infections or abiotic stress, which showed increased intracellular expression of natterin (Dang et al., 2017; Cabrales-Orona et al., 2022).

Zebrafish encode multiple Natterin-like proteins, including the resolved pore-forming toxin NP_001013322.1, encoded by aep-1 (Jia et al., 2016), and loc795232 (Seni-Silva et al., 2022). Our previous work demonstrated that Natterin loc795232 is essential for coordinating immune responses against *Salmonella Typhimurium* (ST). Its absence in natterin (loc795232) knockout (KO) embryos generated by CRISPR/Cas9 increased mortality, impaired locomotor responses, and abrogated inflammasome activation, as measured by caspase-1 activity and IL-1β release, suggesting a role beyond direct pathogen lysis (Pinto et al., 2025).

In mammals, ST infection is controlled through NAIP/NLRC4 and NLRP3 inflammasome activation, leading to caspase-1/11–dependent pyroptosis (Gram et al., 2021). Although zebrafish lack the NAIP/NLRC4 complex, they mount a robust defense involving IL-1β, type I interferon (IFN-I), and inflammasome genes (Stockhammer et al., 2009; Vincent et al., 2016; Widziolek et al., 2021; Levraud et al., 2019).

Intriguingly, emerging evidence across vertebrates reveals intricate bidirectional regulation between IL-1β and IFN-I pathways. IL-1β can induce self-DNA release, activating the cGAS-STING-IFN-I axis (Härtlova et al., 2015), while IFN-I is required for non-canonical inflammasome priming and caspase-11 activation via guanylate-binding proteins (Gbps) (Swanson et al., 2017; Yi, 2020). This reciprocal regulation suggests an amplification loop critical for effective immunity.

Given that Natterin is required for NLRP3-IL-1β responses and considering the interplay between IL-1β and IFN-I, we hypothesized that Natterin-mediated protection against ST depends on IFN-I signaling, thereby serving as a molecular link between inflammasome activation and interferon responses. To test this, we first used wild-type embryos treated with IFN-I neutralizing antibody, followed by the use of CRISPR/Cas9 natterin KO embryos and integrated approaches—including RT-qPCR, Western blotting, immunohistochemistry, and behavioral assays—to dissect the role of Natterin in IFN-I–dependent inflammasome activation during ST challenge.

Our results demonstrate that Natterin is indispensable for mounting a coordinated innate immune defense. We found that CRISPR/Cas9 depletion of natterin (loc795232) abrogates the transcriptional response, leading to the downregulation of *irf3*/*irf7*, the interferon receptor *crfb1*, and the critical interferon-stimulated gene gbp4. This failure in interferon signaling cascades resulted in the impaired maturation of Caspy2 and the downregulation of the pore-forming executioners *gsdme-a/b*, effectively disrupting non-canonical inflammasome activation. The critical role of IFN-I signaling was independently confirmed by neutralizing IFN-I in wild-type embryos, which likewise abolished the protein-level localization of IL-1β and IFN-β, mirroring the phenotype of the natterin-deficient zebrafish.

Collectively, our findings support a novel model wherein Natterin acts as a central upstream regulator that bridges the interferon and inflammasome pathways in teleosts. By establishing Natterin as a key inducer of IFN-φ1 production necessary for non-canonical inflammasome activation, our work provides a robust foundation for understanding this immune amplification circuit.

## 2 Materials and methods

### 2.1 Zebrafish husbandry

Adult zebrafish (<18 months old) from the AB strain (International Zebrafish Resource Center, Eugene, OR, USA) were kept separated by sex and bred under standard conditions of temperature at 28°C, pH 7, and a light-dark cycle (14/10h) in individual aquariums in an ALESCO (Campinas, Brazil) rack using system water (60 μg.ml^−1^ Instant Ocean sea salts). The experiments were carried out under the laws of the National Council for Animal Experiment Control (CONCEA) and approved by the Butantan Institute’s Ethics Committee on the Use of Animals (CEUAIB #4.335.230.323 and #8.197.271.123). The fertilized embryos checked in the Leica EZ4W stereomicroscope (Leica Microsystems, Cambridge, UK) were transferred to 100× 25 mm plastic dishes (#89107-632, VWR) containing 0.5× E2 medium (7.5 mM KH_2_PO_4_, 2.5 mM Na_2_HPO_4_, 15 mM NaCl, 0.5 mM KCl, 1 mM MgSO_4_.7H2O, 1 mM CaCl_2_._2_H_2_O, 0.7 mM NaHCO_3_, and classified according to Kimmel et al. (1995).

### 2.2 Zebrafish anesthesia, dechorionation, and euthanasia

Anesthesia was performed by immersing embryos in 2 ml of 0.5× E2 medium containing 0.4% tricaine (ethyl-3-aminobenzoate, #MS-222, Sigma Chemical Co., St. Louis, MO, USA) for 2 min at room temperature before analysis. At the end of experiments, euthanasia was obtained by immersion in 4% tricaine diluted in 0.5× E2 medium. After exposure, dead embryos were checked in an M205C stereomicroscope (Leica Microsystems, Wetzlar, Germany) to ensure the absence of heartbeat and movement (as proof of mortality). When suitable, 1 dpf (day post-fertilization) embryos were anesthetized and dechorionated by immersion in pronase (#P5147, Sigma) at 0.02 mg.ml^−1^ for 5 min.

### 2.3 CRISPR gRNA-Cas9 of Natterin gene

Depletion of the natterin loc795232 (ID: 795232, XM_017356964.2, chromosome 7) was done according to our group (Seni-Silva et al., 2022) using natterin crRNA (#WD0747994) purchased from Sigma-Aldrich, Burlington, United States. Duplex gRNA was prepared by mixing equal amounts of 50 ng/µl crRNA and 50 ng/µl transactivating crRNA (tracrRNA) (#TRACRRNA05N, Sigma-Aldrich). The gRNA was mixed with Cas9 enzyme, at 250 ng/µl (#CAS9PROT, Sigma-Aldrich), to make ribonucleoprotein (RNP) complex. Embryos of 0 hpf (hours postfertilization) or one-cell stage (*n* = 100 per group) mounted into an agarose-coated plate groove (#16500100, Invitrogen, Carlsbad, California, EU) were injected with a microneedle (#5242952008 femtotips 930000043 with 0.5–0.7 µm Eppendorf, Hamburg, Germany) coupled to a micromanipulator Injectman^®^ 4 Pneumatic PicoPump microinjector (Eppendorf, Hamburg, Germany) pressurized with approximately 2 or 3 nl in the cell using micrometer-scale 1 mm with 0.01 mm divisions for calibration (#2280-13-1EA, Ted Pella). After injection, live F0 crispants, henceforth called knockout (KO), distinguished due to their hypopigmentation phenotype (Burger et al., 2016; Hoshijima et al., 2019), were incubated in a petri dish in 0.5× E2 medium at 28°C, accompanied by wild type (WT) only injected at 0 hpf with 1× Danieau’s buffer (58 mM NaCl, 0.7 mM KCl, 0.4 mM MgSO4, 0.6 mM Ca(NO3)2, 5.0 mM HEPES; pH 7.6) containing 1% phenol red (#P3532, Sigma). The expression of the natterin mRNA was confirmed in the whole-body embryo by RT-qPCR.

### 2.4 Whole-mount in-situ hybridization (WISH) for detection of the Natterin gene

The 1 dpf embryos microinjected with CRISPR/Cas9 (KO) or non-injected (WT) were fixed in fresh 4% formaldehyde overnight at 4°C. Then, the embryos were dehydrated in 100% methanol and stored at −80°C. Embryos were removed from the ultrafreezer and rehydrated with gradient dilutions of methanol in PBS (75%, 50%, and 25%). The embryos were digested with 10 μg.ml^−1^ of proteinase K (#P6556, Sigma-Aldrich) in 200 μl of proteinase K buffer (0.005 M Tris-HCl, 0.001 M EDTA, 0.001 M NaCl in RNase-free water) at room temperature for 30 min and then rinsed in 4% formaldehyde for 20 min to stop the digestion, followed by four rounds wash with PBST [1× PBS, 0.1% Tween 20 (v/v)] to remove the formaldehyde residues. Later, they were incubated in 200 μl of complete hybridization buffer (50% formamide in 5× SSC buffer, 0.1% tween 20, 500 μg.ml^−1^ yeast RNAse (#AM9789, Ambiam), and 50 μg.ml^−1^ heparin (#CC-4396A, Lonza), pH 6) for 3h at 70°C. After, samples were incubated overnight with 40 nM of the DIG-labeled natterin gene detection probe (#339500 LCD0168623 BKG LOC795232_1, miRCURY LNA miRNA, Qiagen Venlo, Netherlands) at 70°C. The next day, the probe was removed by washing with incomplete (without heparin or RNAse) hybridization buffer in 2× SSC (75%, 50%, and 25%) and incomplete hybridization buffer in 0.2× SSC (75%, 50%, and 25%) for 10 min each at room temperature. Then, the embryos’ non-specific antibody sites were blocked with blocking solution (1× PBST, 2% tilapia serum, and 2 mg.ml^−1^ BSA) for 3.5h at room temperature. Anti-DIG-AP (#11093274910, Roche Diagnostics, Basel, Switzerland) at 1:300 dilution in blocking solution was added and agitated (40 rpm) overnight at 4°C. The embryos were washed with PBST six times for 15 min at room temperature. Then, the embryos were soaked in 200 μl of fresh staining solution prepared with 50 mg.ml^−1^ BCIP (5-Bromo-4-chloro-3-Indolyl phosphatase; #11383221001, Roche Diagnostics) and 100 mg.ml^−1^ NBT (4-nitro blue tetrazolium chloride; #11383213001, Roche Diagnostics) in the dark at room temperature for 4h, monitored in a stereomicroscope every 1h. The colorimetric reaction was stopped by washing the embryos three times in the stop solution (1× PBS, 1 mM EDTA, and 0.1% Tween 20, pH 5.5) and fixed with 200 μl of 100% glycerol overnight under agitation (40 rpm) at room temperature. Embryos were visualized on AxioVision^®^ software (Carl Zeiss, Oberkochen, DE) in 60 and 100× magnification. The qualitative expression of the natterin gene was confirmed in the entire larva by an intense blue-purple precipitate signal (Seni-Silva et al., 2022).

### 2.5 Bacterial stimulation by immersion bath and pharmacological treatments

Previously dechorionated 1 dpf WT or KO zebrafish embryos (*n* = 25/group, quadruplicate) were stimulated by immersion in fresh 0.5× E2 medium with heat-killed Salmonella enterica sorovar Typhimurium (henceforth called ST) (#TLRL-HKST2, CDC6516-60, Invivogen) at 106 cells/ml for 2h. Independent groups of 1 dpf embryos (*n* = 60) were previously neutralized by immersion in fresh 0.5× E2 medium for 30 min with the monoclonal mouse IgG2a anti-human IFN-β neutralizing antibody (clone 13A2, #mabg 2 hifnb-3, Invivogen) at 2 µg/ml and then exposed or not by immersion to ST for 2h, according to Habjan et al. (2024). Pretreated embryos with the same amount of IgG2a isotype (mouse IgG2a control clone T9C6 #mabg2a-ctrlm, Invivogen, San Diego, United States) were considered control. For the Natterin protein detection experiment by WB, independent groups of embryos were pretreated for 1h with 1 μg/ml Pam3CSK4 (#2201, Imgenex, Bhubaneswar, India), a TLR2/TLR1 agonist that primes NF-κB–dependent inflammasome transcription, or 1 μM MCC950 (#17510, Cayman), an ATPase inhibitor blocking NLRP3-ASC oligomerization, both diluted in fresh 0.5× E2 medium before stimulation for 2h with ST. Embryos that remained in fresh 0.5× E2 medium without stimulation or treatments were considered a negative-control group. All groups were incubated at 28°C and analyzed for mortality. In addition to mortality, embryos were evaluated for sublethal abnormalities such as head/eye malformation, abnormal yolk sac absorption, edema in the pericardium or yolk sac, non-inflated swim bladder, and abnormal pigmentation; and regarding teratogenic abnormalities such as curved tail, shortened tail, scoliosis, and delayed growth through visualization of the embryos photographed side by side and aligned by the head using a Leica M205C stereomicroscope.

### 2.6 Zebrafish locomotor behavior assessment

Locomotor activity was investigated by analyzing the swimming behavior after 72h post stimulation upon dark–light transition according to the modified method of Scott et al. (2016). ST-stimulated or negative-control (*n* = 20/group) were transferred to 96-well plates, with one larva per well in 100 μl of 0.5× E2 medium, and analyzed in a Zebrabox System (ViewPoint Life Sciences, Lyon, France). Embryos were analyzed for a total of 32.5 min, consisting of 30 min of acclimatization in the light (Lux: 12%) followed by five cycles of 1 s in the dark (Lux: 0%) and 29 s in the light (Lux: 12%). Locomotor activity was quantified and analyzed using ZebraLab™ version 3.52 by ViewPoint. The mean speed was set to values between 1.8 and 4.0 mm/s, while any movement slower than 1.8 was considered inactivity and above 4.0 as agitated behavior. The total distance results were obtained by summing the distances moved while at medium and agitated speeds, and the total average speed by dividing the distance by the analysis time.

### 2.7 SDS-PAGE electrophoresis

Zebrafish embryos (*n* = 80/group) from the different experimental groups were macerated in 500 µl of western blot lysis buffer (WB lysis buffer: RIPA, #9806 Cell Signaling, added with protease and phosphatase inhibitor with EDTA (#88668, Thermo) using a 10-ml syringe with a 1-mm needle. The recovered lysates were left in liquid nitrogen until freezing. After thawing, they were centrifuged at 14,000 rpm at 4°C for 10 min to recover the supernatants containing proteins that were concentrated by precipitation in acetone for 12h at -20°C. The protein precipitate was solubilized in 200 µl of Milli-Q water for subsequent determination of the protein content using the Bradford method. Samples containing 5 µg of protein in a volume of 39 µl were incubated with sample buffer (Novex 4×, #B0007, Novex, Waltham, United States) and reducing agent (Novex 10×, #B0009, Novex) for 10 min at 70°C and applied to Novex Wedgwell 8%–16% Tris-glycine 10W gel (#XP0816BOX, Thermo) in Bolt Mini Gel Tank system (#B4477599, Novex, Thermo) and source (#EPS601, G&E) with running standard: 40 min under 165 V, 200 mA, and 100 W in MES buffer (Novex 20×, #B0002, Novex). The molecular mass standards ladders (SeeBlue Plus2 Pre-Stained Standard NuPAGE MES, Novex, #LC5925, Thermo) used were myosin 188 kDa, phosphorylase 98 kDa, bovine serum albumin 62 kDa, glutamate dehydrogenase 49 kDa, alcohol dehydrogenase 38 kDa, carbonic anhydrase 28 kDa, red myoglobin 17 kDa, lysozyme 14 kDa, aprotinin 6 kDa, and insulin 3 kDa.

### 2.8 Western blot

After running, proteins were transferred to nitrocellulose membrane (#RPN203D, Hybond, Amershan) using the traditional Mighty Small Transfer method (#80620426, G&E), immersed in 1X WB transfer buffer for 2h. The bands were identified by Ponceau solution staining (Sigma, #P3504), and the membrane, previously washed with TBST solution, remained overnight in blocking solution (TBST-5% powdered milk). The specific proteins were detected in the iBindTM Flex Western System using iBind Flex Cards (Invitrogen, #SLF2010) and the solution made with the iBind solution Kit (Invitrogen, #SLF1020) and the primary antibodies: rabbit polyclonal IgG anti-mouse caspase-1 (p-10, M-20) (#sc-514, Santa Cruz Biotechnology, Dallas, United States, 1/200, detects the immature form with 50 kDa and the mature form around 28 kDa), rabbit polyclonal IgG raised against amino acids 301–350 of caspase-11 of mouse origin (p-10 (M-50; #sc-28231; Santa Cruz Biotechnology, 1/200, detects the immature form with 50 kDa and the mature form around 28 kDa) followed by rabbit HRP-labeled anti-rabbit IgG TrueBlot second antibody (Rockland, #18-8816–33 at 1/1,000) for 3h. Natterin was detected with rabbit anti-serum against natterin purified from Thalassophryne nattereri venom (dimeric form around 62 kDa; dilution: 1/50), followed by the second antibody anti-rabbit IgG HRP TrueBlot (#18-8816-33, Rockland; dilution 1/1,000). Mouse monoclonal IgG1κ anti-mouse pan-actin clone C4 (#MAB-1501, Merck, Darmstadt, Germany; 1/3,000–43 kDa, 1/3,000) followed by anti-mouse IgG HRP (#1170-05, Southern Biotech; 1/500) was also used. The revelation was made by the addition of SuperSignal West Femto Maximum Sensitivity Substrate (#34095, Thermo Fisher, Waltham, United States). Using the Amersham Imager 680 photodocumentator, with an exposure time of 1 s, the chemiluminescent bands were detected. Bar graphs corresponding to protein expression levels were generated as a percentage increase relative to the control group.

### 2.9 Mature IL-1β and IFN-I analysis by immunohistochemistry

Whole embryos from the different groups (*n* = 6) were rinsed with PBS, fixed in 4% formaldehyde in PBS overnight, and then dehydrated by washing them sequentially with 30%–70% methanol (MeOH) diluted in PBST (50 ml 10× PBS, 1 ml 10% Tween 20, volume up to 500 ml with dH_2_O) for 10 min each at room temperature (RT) with stirring. After, embryos were rehydrated by washing them sequentially with 70%–30% MeOH diluted in PBST for 10 min each at RT, with two sequential 5-min washes with PBST. After removing the PBST, embryos were transferred to a 30% sucrose solution diluted in deionized water and kept overnight. For cryosection, fixed embryos were mounted (three embryos per cassette/total two cassettes) in OCT compound (#Neg-50, Richard Allan), sectioned at 18 μm in a coronal position on a cryotome (Cryostat Leica CM1860), and processed for identification by IHC. Slides left in a dark chamber were permeabilized (PBS + 10% Triton 100) for 1h and blocked (PBS + 0.1% Triton 100 + 10% BSA) overnight at 4°C. They were then incubated overnight at 4°C with the polyclonal rabbit HRPO-labeled IgG anti-rainbow trout IL-1β antibody that recognizes the rainbow trout mature IL-1β peptide with 20 kDa according to Hong et al. (2001) (#CLF016HP, Cedarlane, 1/3,000) or the polyclonal rabbit HRPO-labeled IgG anti-rainbow trout IFN-β antibody (#CLF005HP, Cedarlane, 1/3,000) according to Altmann et al. (2003). After a new washing cycle, 3’3’ diamonibenzidine (DAB) chromogenic solution (#D4168, Sigma) was added with H_2_O_2_ for 5 min in the dark. Sections were counterstained with hematoxylin. Images were obtained with the Axio Imager A.1 microscope (Carl-Zeiss, Germany) coupled to the Zeiss AxioCam HRc camera with 10×, 20×, or 40× objectives. Each experimental group formed by two cassettes containing three embryos each, generated two slides containing eight sections that, after staining, were analyzed to count positive cells using an image processing package (FIJI ImageJ).

### 2.10 RNA extraction and cDNA synthesis

Zebrafish embryos (*n* = 100/group) were ground in 300 µl of Trizol (#15596026, Thermo Life Technologies, Waltham, United States) and frozen at −80°C before the next step. RNA extraction was done following classical protocol. Briefly, each frozen sample tube was thawed and kept at 4°C for 10 min before centrifugation at 12,000*rpm* for 15 min at 4°C. 200 μl of chloroform (#C7559, Sigma) was then added to each supernatant, vigorously mixed, and incubated at 28°C–30°C for 5 min, followed by centrifugation at 4°C, 12,000*rpm* for 15 min. The upper aqueous phase was transferred to a new tube, and 1 μl glycogen (#10814010, Invitrogen) and 700 μl isopropanol (#I9516, Sigma) were added and mixed well. Samples were incubated at −20°C overnight for RNA precipitation. After thawing, samples were centrifuged at 4°C, 12,000*rpm* for 15 min, the supernatant was discarded, and the RNA pellet was washed with 700 μl of cold 75% ethanol twice and centrifuged at 4°C, 12,000*rpm* for 15 min. After samples were air-dried for 10 min, and they were resuspended in 50 μl of RNase-free water (#10977-015, Invitrogen).

After suspension, samples were centrifuged twice, and the supernatant was used for subsequent exclusion of residual DNA with the Turbo DNA-free kit (#AM1907, Invitrogen), according to the description of Chomczynski and Sacchi (1988). Then, 0.1 μl of 10× Turbo DNase™ Buffer and 1 μl of Turbo DNase™ Enzyme were added to the RNA, then mixed gently, and then incubated at 37°C for 20 min. 5 μl of DNase Inactivation Reagent was added and incubated at 37°C for 5 min. After centrifugation at 4°C and 10,000*rpm* for 2 min twice, the supernatant containing the RNA was quantified using NanoDrop spectrophotometer (260/280 nm). cDNA synthesis was performed using one microgram of total RNA to a final 20 μl of SuperScript™ Vilo™ cDNA Synthesis Kit (#11754050, Life Technologies, Waltham, United States). Tubes were incubated in the thermocycler (VeritiPro Thermal Cycler, Thermo Fisher Scientific, Waltham, United States) at 25°C for 10 min, 42°C for 60 min, and at 85°C for 5 min and stored at –20°C until use.

### 2.11 Gene expression analysis by RT-qPCR

RT-qPCR was performed using the real-time PCR system (QS3-Quanti Studio 3 Real Time PCR Systems, Thermo Fisher Scientific), and each reaction included 2 μl of cDNA diluted 1/20, the primer set (2 μl), and 4 μl of Power SYBR™ Green PCR Master Mix (#4367659, Life Technologies). Primers (Thermo Invitrogen Technologies, Waltham, United States) were designed to amplify caspy2, gbp4, gbp1, *gsdme-a*, *gsdme-b*, *irf3*, *irf7*, myd88, sting, *crfb1*, crfb2, casp-3, and casp-8 as described below (**Table 1**). The PCR protocol included the following parameters: hot start at 50°C for 2 min and 95°C for 10 min, 40 cycles of 95°C for 15 s, 60°C for 1 min and 95°C for 15 s, and melt curve of 60°C for 1 min and 95°C for 15 s. For PCR products more than 200 bp, the protocol included a hot start at 50°C for 2 min, and 94°C for 10 min, 40 cycles of 94°C for 15 s, 58°C for 2 min and 95°C for 15 s, and a melt curve of 58°C for 1 min and 95°C for 15 s. All samples were run in triplicate. Relative gene expression was determined by comparing β-actin-1 endogenous control using the 2−ΔΔCt method according to Livak and Schmittgen (2001), and values were expressed as fold change relative to the expression level in the unstimulated WT group at 0 hpf.

**Table 1:** Primer specifications.

Gene	Sequence (5’->3’)	Specifications	Product size	Localization
β-actin-1-F	TGCTGTTTTCCCCTCCATTG	20 nt; 50% GC; Tm: 65°C	66	184-204
β-actin-1-R	TTCTGTCCCATGCCAACCA	19 nt; 53% GC; Tm: 67°C		250-231
loc795232-F	CAGGTTCTCCCTTTTCATTTACTGG	25 nt, 44% GC, Tm: 65°C	486	104-128
loc795232-R	TGATCTCTTCCACTGCCACC	20 nt, 55% GC, Tm: 67°C		589-570
gbp1-F	GTGGAGGGTAGGAGAGCTTCG	21 nt; 62% GC; Tm: 61,63 °C	97	114-135
gbp1-R	GGGCGAAGGCATCATTGTGG	20 nt; 60% GC; Tm: 62,01 °C		210-190
*crfb1*-F	TGCAATCCCTGCTCCTGTCAA	21 nt; 52% GC; Tm: 62 °C	143	330-351
*crfb1*-R	TCAAATGCTCTGTTTGCTTTCCCT	24 nt; 42% GC; Tm: 62 °C		472-448
crfb2-F	CCGAATGCGTCCTATACCCC	20 nt; 60% GC; Tm: 60 °C	200	531-551
crfb2-R	CCCAACCCTTCCTCTGTTCCA	21 nt; 57% GC; Tm: 62 °C		730-709
gsdmea-F	TGCTTTTGTGCACTGGCAGA	20 nt; 50% GC; Tm: 61 °C	130	992-1012
gsdmea-R	CGTCACTGCTCAGGGTTTCG	20 nt; 60% GC; Tm: 61 °C		1121-1101
*irf3*-F	CAAAACCGCTGTTCGTGCC	19 nt; 58% GC; Tm: 61 °C	141	266-285
*irf3*-R	CATCGTCGCTGTTGGAGTCCT	21 nt; 57% GC; Tm: 62 °C		406-385
*irf7*-F	AGGCAGTTCAACGTCAGCTACCAT	24 nt; 50% GC; Tm: 64 °C	97	424-448
*irf7*-R	TTCCACAAGTTGAGCAATTCCAG	23 nt; 44% GC; Tm: 60 °C		520-497
gsdmeb-F	GCACCATCACTGAGGAGGTTC	21 nt, 57% GC, Tm: 61 °C	115	640-661
gsdmeb-R	TGTCGTACGCAATTGAAGGCT	21 nt, 48% GC, Tm: 61 °C		753-732
casp8-F	CAAGGGCAAAGCTGGGAAGA	20 nt, 55% GC, Tm: 61 °C	146	524-544
casp8-R	CCTCTCGATCATAACAGCCAGC	22 nt, 48% GC, Tm: 61 °C		669-647
caspyb-F	CAGAACGAACGTGCAAAGCG	20 nt, 55% GC, Tm: 61 °C	96	263-283
caspyb-R	GATGGGCTGCGGTTCTTCAG	20 nt, 60% GC, Tm: 61 °C		358-338
casp3-F	AAAGGATCCCAGTGGAGGCAGATT	24 nt; 46% GC; Tm: 62 °C	159	850-874
casp3-R	TGGTCATGATCTGCAAGAGCTCCA	24 nt; 50% GC; Tm: 64 °C		1008-984
gbp4-F	GCTGGCTGGGAAACGAACA	19 nt, 58% GC, Tm: 61 °C	96	450-469
gbp4-R	GCTTTAGTGGGATGGGGCAC	20 nt, 60% GC, Tm: 61 °C		545-525

### 2.12 Statistical analysis

All experiments were performed in triplicate to establish proof of concept, followed by additional biological replicates with varying numbers of embryos to achieve adequate sample sizes for each analytical approach. Data are presented as mean ± standard error of the mean (SEM). For parametric data, we performed one-way analysis of variance (ANOVA) with Bonferroni *post-hoc* correction for multiple comparisons, comparing the mean of each column with the mean of every other column. In all analyses, results were considered statistically significant at *p* < 0.05. All statistical tests were conducted using GraphPad Prism software (version 6.02; GraphPad Software, La Jolla, CA, USA).

## 3 Results

### 3.1 The control response against ST is IFN-I-dependent

**Figure 1:**
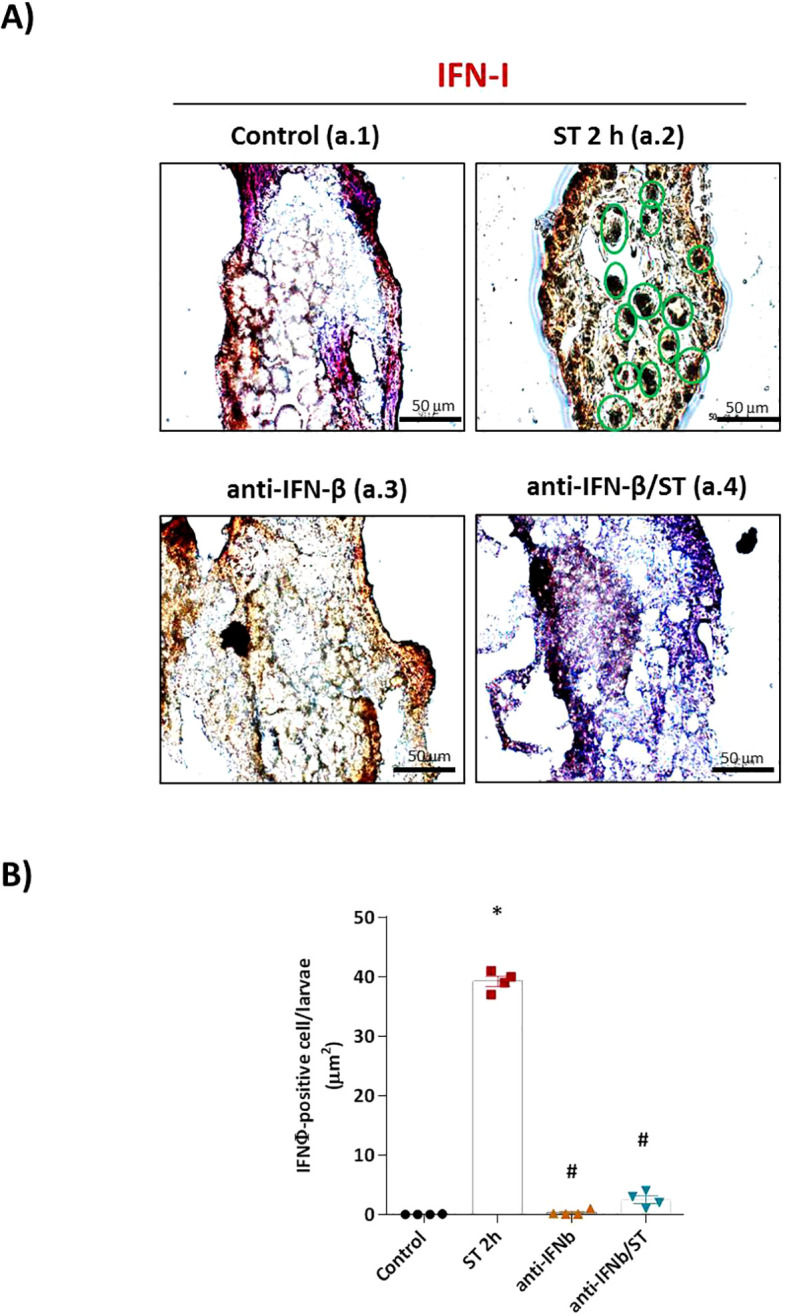
IFN-φ neutralization abolishes ST-induced interferon production in zebrafish embryos. **(A)** Representative immunohistochemical staining of IFN-φ (brown DAB precipitate) in 1 dpf wild-type (WT) embryos under four conditions: (a.1) unstimulated control, (a.2) ST stimulation (10^6^ cells/ml for 2h), (a.3) anti-IFN-β antibody pretreatment alone, and (a.4) anti-IFN-β pretreatment followed by ST stimulation. Scale bar: 50 μm. **(B)** Quantification of IFN-φ-positive cells from two slides per group (eight sections/slide, three embryos/section) using Fiji-ImageJ. Data represent mean ± SEM (*n* = 3 independent experiments); *p < 0.05 versus unstimulated control WT, #*p* < 0.05 versus ST-stimulated WT.

To define the role of type I interferon (IFN-I) in the zebrafish defense against ST, we first neutralized IFN-φ signaling using a specific antibody prior to challenge. Our analysis revealed that IFN-I is indispensable for an effective immune response. Immunohistochemistry using an anti-rainbow trout IFN-β antibody, which cross-reacts with zebrafish IFN-φ, showed intense IFN-φ production in ST-stimulated embryos (**Figures 1A-a.2**), which was completely abolished by pre-neutralization (**Figure 1A-a.4**, quantified in **Figure 1B**). No signal was detected in unstimulated controls or embryos treated with the neutralizing antibody alone (**Figures 1A-a.1, a.3**).

**Figure 2:**
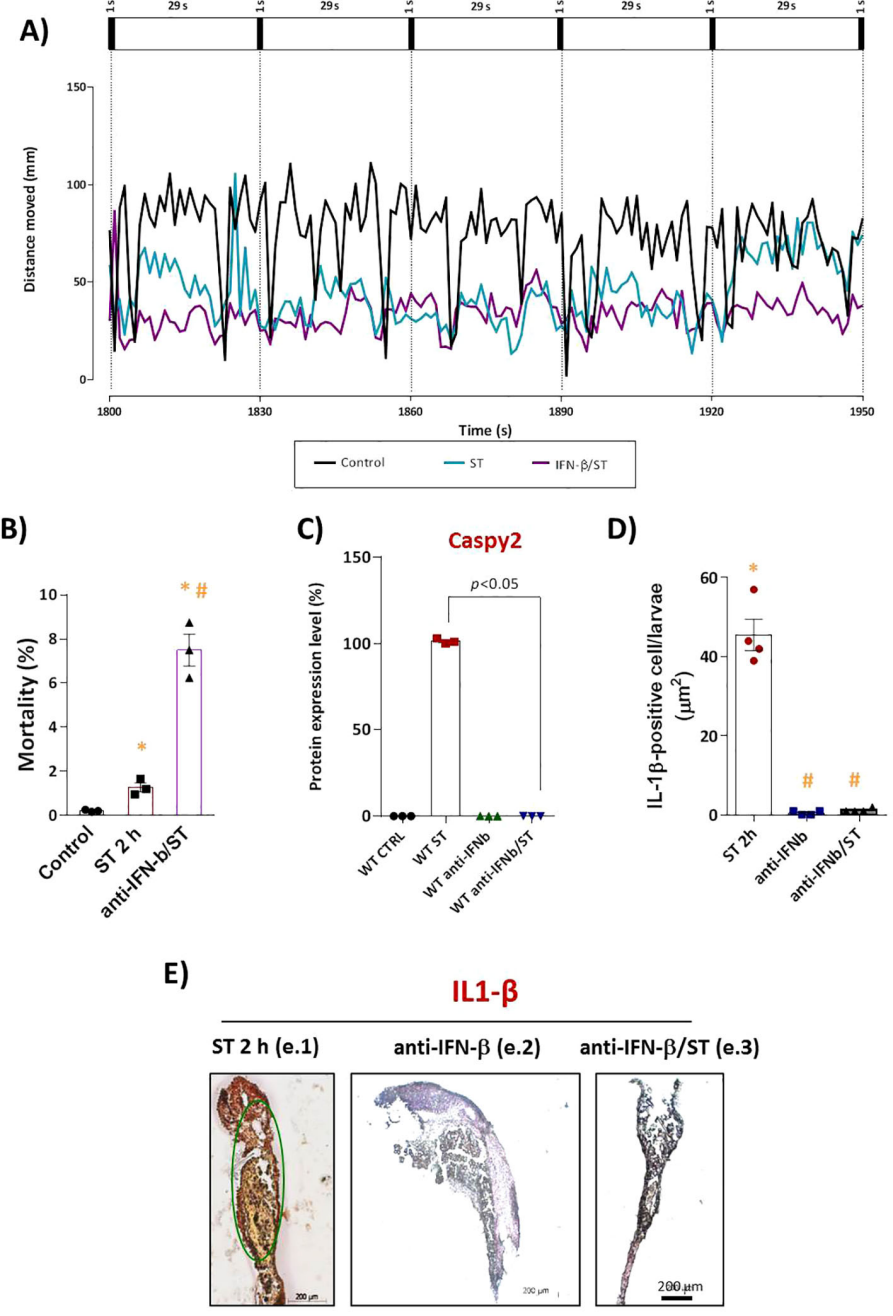
IFN-I signaling is essential for IL-1β–mediated protection against ST stimulation. **(A)** Locomotor activity (total distance traveled) assessed 72h after stimulation at 28°C. Mortality count **(C)** and Caspy2 expression in WT embryos after ST stimulation (10^6^cells/ml, 2h) with or without anti-IFN-β antibody pretreatment. The corresponding bar graph **(D)** represents the protein expression levels as a percentage of the control. **(E)** Quantification and **(F)** representative IHC images of mature IL-1β (mIL-1β)–positive cells (brown DAB precipitate) in: (f.1) ST-stimulated embryos, (f.2) pretreatment with anti-IFN-β alone, and (f.3) pretreatment with anti-IFN-β followed by ST stimulation. Scale bar: 200 μm. Data represent mean ± SEM (*n* = 3 independent experiments); *p < 0.05 versus unstimulated control WT, #*p* < 0.05 versus ST-stimulated WT.

Functionally, this blockade of IFN-φ signaling had severe consequences. Neutralization resulted in a sixfold increase in ST-induced mortality compared to ST-stimulated controls (**Figure 2B**), with no significant mortality in control groups. Furthermore, IFN-φ neutralization exacerbated ST-induced phenotypic alterations, including increased depigmentation and developmental delay (data not shown). Locomotor activity assays revealed that the ST challenge alone induced significant hypoactivity. This behavioral impairment was dramatically amplified in IFN-φ-neutralized embryos (**Figure 2A**), indicating that IFN-φ signaling is crucial for mitigating the pathogenic effects of ST.

At the molecular level, Western blot analysis demonstrated that the robust expression of mature Caspy2 protein induced by ST challenge (**Supplementary Figures S1A, B**–lane 2) was drastically reduced in embryos where IFN-φ was neutralized prior to challenge (lane 4). Levels remained low in uninfected controls and antibody-only groups (lanes 1 and 3), a finding supported by quantitative densitometric analysis (**Figure 2C**).

Given the established crosstalk in mammals where IL-1β drives IFN-I production and IFN-I reciprocally enhances inflammasome activity; we investigated this loop in our model. We found that neutralizing IFN-φ also abolished the release of mature IL-1β (mIL-1β) in response to ST challenge (**Figures 2D, E**). This result demonstrates that IFN-φ is not only a downstream effector but also a critical upstream regulator of IL-1β maturation, revealing an essential positive-feedback loop in the antibacterial innate immune response of zebrafish.

### 3.2 Natterin is essential for the transcriptional activation of the IFN-I pathway

**Figure 3:**
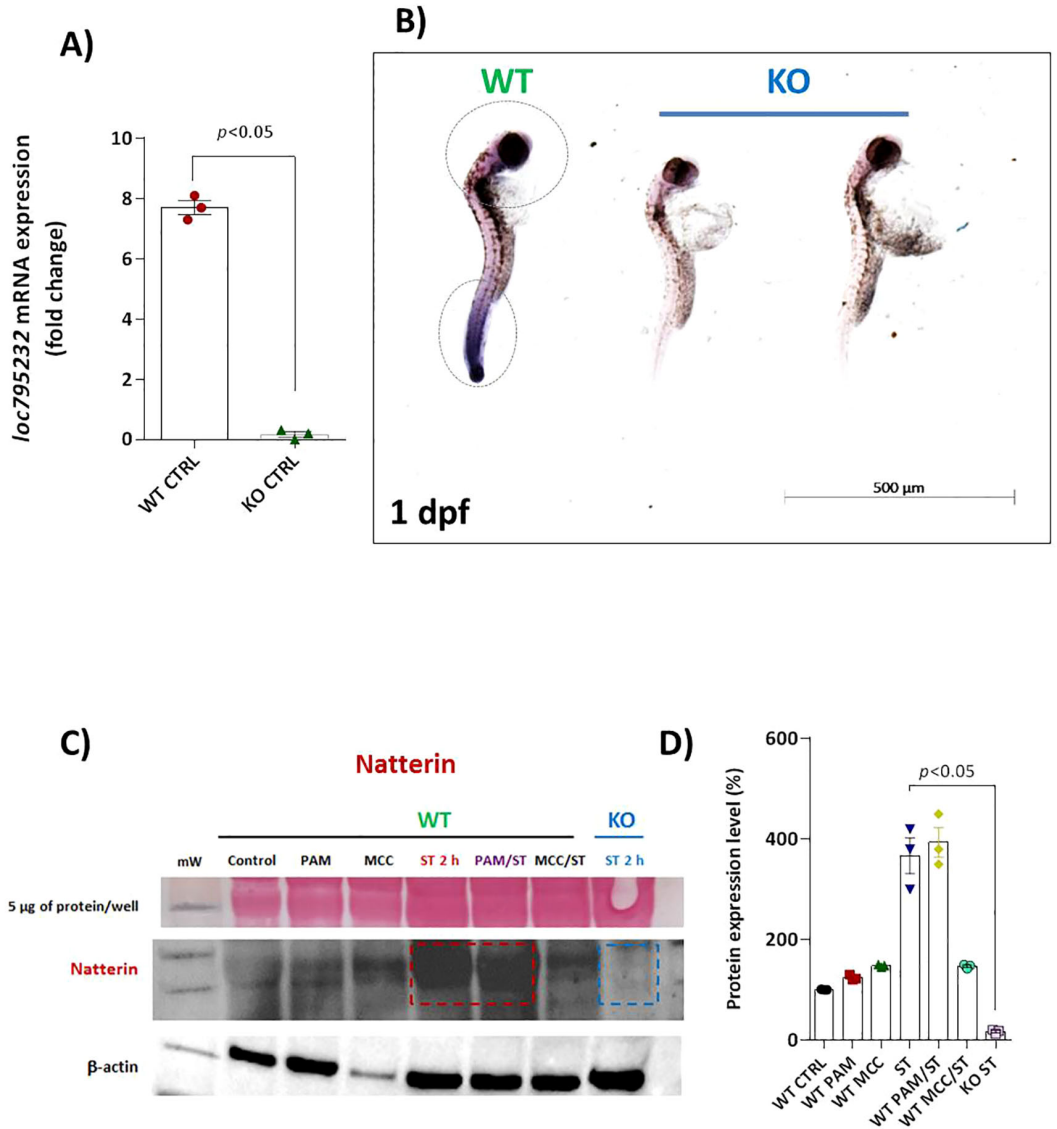
Effectiveness of CRISPR/Cas9 depletion of loc795232. Basal expression of loc795232 mRNA transcripts in unstimulated WT or KO embryos (*n* = 100/group) at 1 dpf by RT-qPCR **(A)** or by *in situ* hybridization **(B)**. All qPCR data normalized to β-actin and expressed as fold change relative to 0h WT unstimulated control. Data represent mean ± SEM of three biological replicates. Independent groups of 1 dpf embryos previously treated or not with Pam3CSK4 or MCC950 and subjected to ST stimulation for 2h, as well as KO larvae stimulated with ST were processed for Natterin detection by WB **(C)** using anti-Natterin serum (62 kDa dimeric form) and anti-IgG TrueBlot HRP. The corresponding bar graph represents the protein expression levels as a percentage of the control **(D)**, and Ponceau S staining is visualized in the first horizontal line.

To investigate the role of Natterin in the innate immune response, we first confirmed the successful depletion of the natterin (loc795232) gene using CRISPR/Cas9. In crispant embryos (KO), RT-qPCR and *in-situ* hybridization revealed a complete absence of Natterin mRNA compared to wild-type (WT) controls (**Figures 3A, B**). This genetic ablation was confirmed at the protein level by Western blot. Natterin protein, detected primarily in its dimeric form (62 kDa), was present at low levels in control groups and was highly expressed in WT embryos stimulated with ST, with or without Pam3CSK4 pretreatment. A faint band was observed in embryos where the NLRP3 inflammasome was inhibited with MCC950 prior to ST stimulation. Critically, no Natterin protein was detected in KO embryos following the ST challenge (**Figure 3C**; **Supplementary Figure S2**), a finding supported by quantitative densitometry (**Figure 3D**), confirming that the genetic depletion completely abolished protein translation.

Having established the KO model, we used RT-qPCR to profile the mRNA expression of key interferon pathway genes. While acknowledging that mRNA levels are a valuable indicator of pathway activation, especially when protein detection is limited, we focused on transcriptional regulation as a primary readout of immune signaling (Liu et al., 2016; Lan et al., 2009; Rassier et al., 2020; Beler et al., 2024; Bustin, 2024).

**Figure 4:**
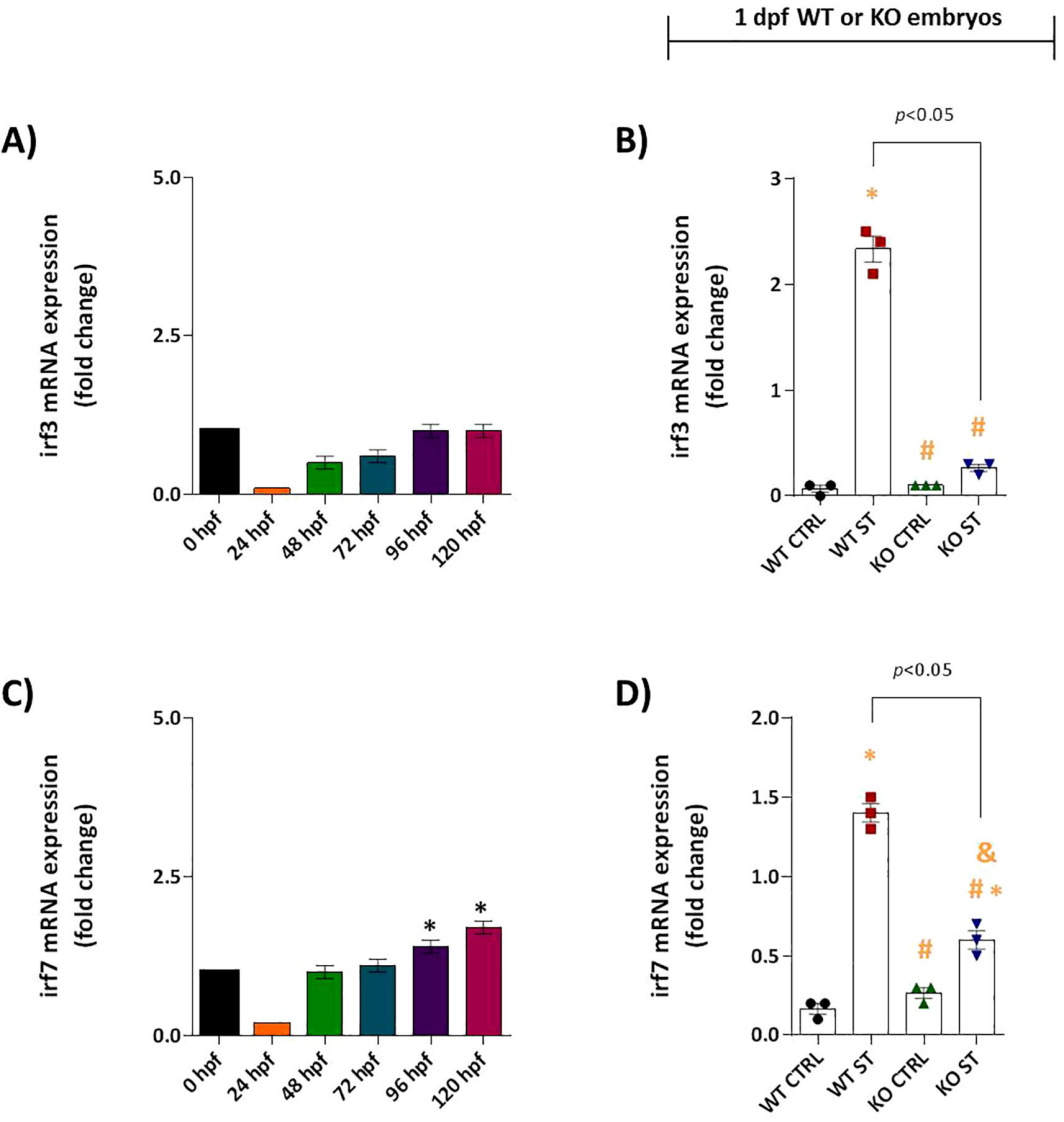
Natterin is required for ST-induced *IRF3*/*IRF7* activation. **(A)** Developmental expression profile of *irf3* in unstimulated WT embryos (*n* = 100/group) from 24–120 hpf. **(B)** *irf3* induction 2h post-ST stimulation in WT versus natterin knockout (KO) embryos. **(C)** Baseline *irf7* expression in unstimulated WT embryos. **(D)** One day post-fertilization ST-mediated *irf7* upregulation in WT and KO embryos. All qPCR data normalized to β-actin and expressed as fold-change relative to 0h WT unstimulated control. Data represent mean ± SEM of three biological replicates; *p < 0.05 versus unstimulated control WT, #*p* < 0.05 versus ST-stimulated WT.

We found that the ST-induced interferon response is profoundly dependent on Natterin. In WT embryos, *irf3* mRNA was not constitutively expressed (**Figure 4A**) but was robustly upregulated upon ST stimulation (2.33 vs 0.06 in control). This induction was entirely abolished in Natterin KO embryos (**Figure 4B**). Similarly, *irf7* exhibited low baseline expression only from 96 hpf onwards in unstimulated WT embryos (**Figure 4C**). The ST-induced overexpression of *irf7* (1.4 vc 0.16 in control) was also strongly suppressed in the absence of Natterin (**Figures 4C, D**). These results demonstrate that Natterin is essential for the transcriptional activation of the key interferon regulatory factors *irf3* and *irf7* during bacterial challenge.

**Figure 5:**
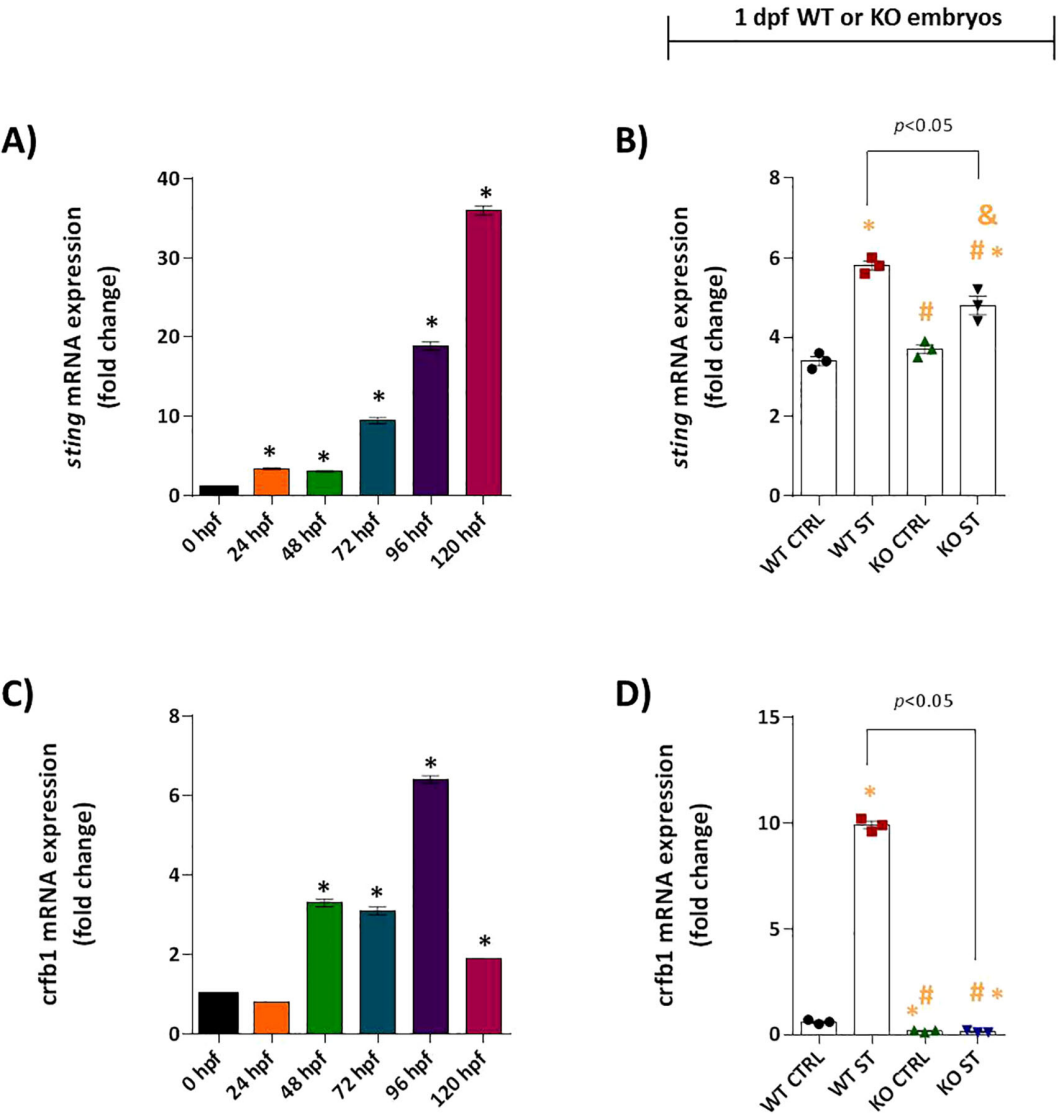
Natterin controls key regulators of type I IFN response. Basal sting **(A)** and *crfb1* **(C)** mRNA expression profiles were analyzed in unstimulated WT embryos (*n* = 100/group) at 24h intervals by RT-qPCR. One day post-fertilization ST-induced sting **(B)** and *crfb1* **(D)** expression in WT and natterin KO embryos was compared. All qPCR data normalized to β-actin and expressed as fold change relative to 0h WT unstimulated control. *p < 0.05 versus unstimulated control WT; #*p* < 0.05 versus ST-stimulated WT.

We next assessed the role of Natterin in regulating upstream adaptor proteins of the IFN-I pathway. Analysis of myd88 revealed a null expression pattern; it was not expressed constitutively and was not induced by ST stimulation in either WT or KO embryos (**Supplementary Figure S5A, B**). In contrast, sting mRNA was constitutively expressed at moderate levels in unstimulated WT embryos (**Figure 5A**) and was further upregulated 1.7-fold by ST stimulation (**Figure 5B**). This induction was partially dependent on Natterin, as KO embryos showed a reduced response (**Figure 5B**). This partial dependence suggests that although Natterin contributes significantly to the upregulation of STING after bacterial challenge, STING is not the sole regulator of IFN-I production.

### 3.3 Natterin is required for Gbp4/Caspy2-mediated non-canonical inflammasome activation

Having established that Natterin is essential for the IFN-I transcriptional response, we next sought to define its role in the downstream signaling events that execute bacterial defense. In zebrafish, type I interferons (IFN-φ) signal through heterodimeric receptor complexes involving CRFB subunits to induce interferon-stimulated genes (ISGs), including guanylate-binding proteins (Gbps), which are critical for non-canonical inflammasome activation (Aggad et al., 2009; Tretina et al., 2019).

Our investigation into the specific IFN-φ receptor revealed a strict dependence on Natterin. The receptor subunit *crfb1* (the ligand for IFN-φ1) was constitutively expressed from 48 hpf in wild-type (WT) embryos, with levels stabilizing by 96 hpf (**Figure 5C**). Crucially, ST stimulation induced a robust 16.5-fold upregulation of *crfb1* in WT embryos. In stark contrast, natterin KO embryos displayed no detectable *crfb1* mRNA, either at baseline or after stimulation (**Figure 5D**), suggesting that Natterin is required for both the constitutive and inducible expression of this key receptor. This near-total absence points to a potential negative feedback loop disrupting the entire IFN-I signaling axis. Conversely, crfb2 (associated with IFN-φ3) was not expressed under any condition in WT or KO embryos (**Supplementary Figures 5C, D**), confirming that the IFN-φ1/*CRFB1* axis is the primary pathway activated in this model.

**Figure 6:**
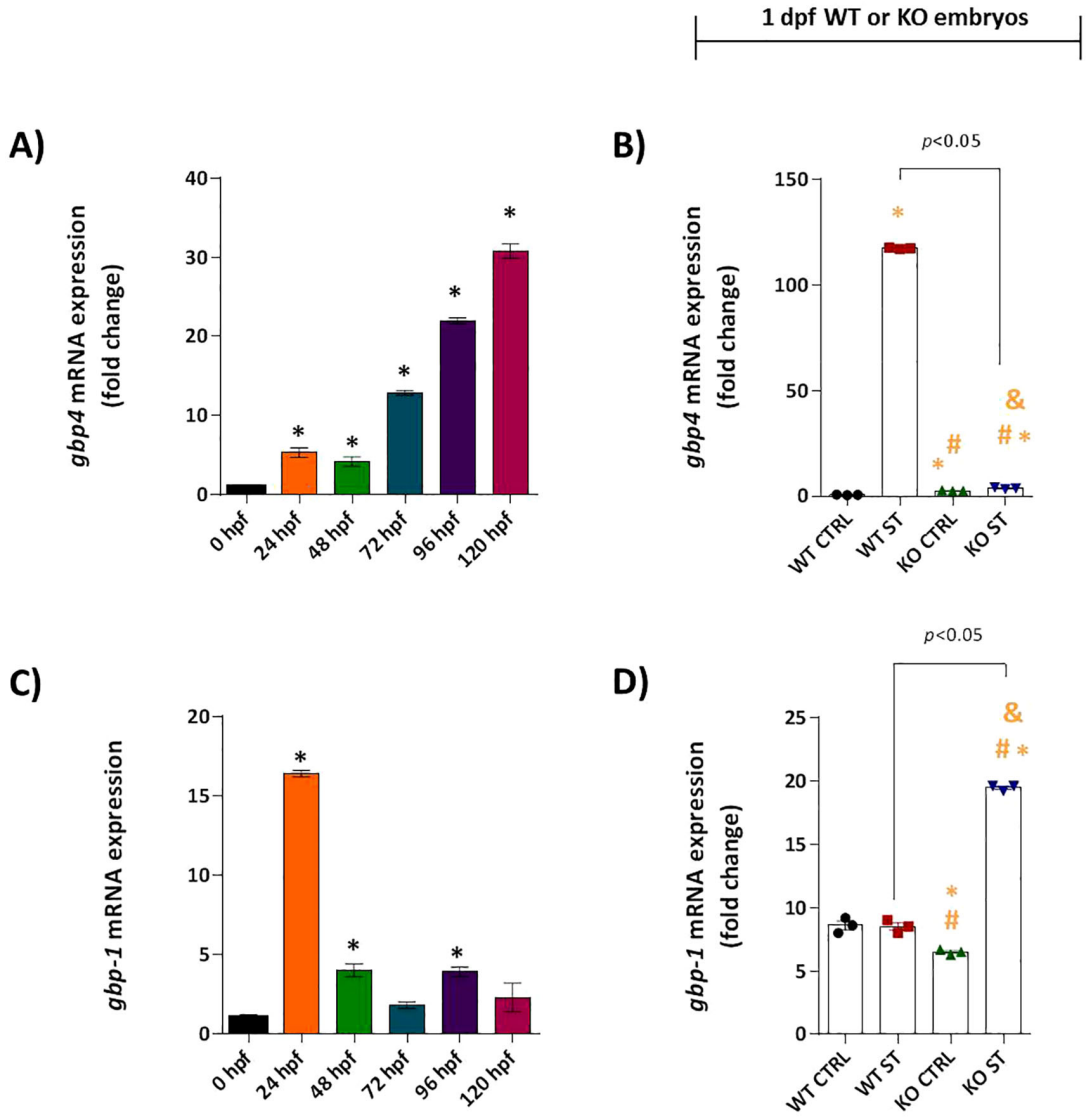
Natterin is required for Gbp4 induction. Constitutive expression of gbp4 **(A)** and gbp1 **(C)**, was analyzed in unstimulated WT embryos (*n* = 100/group) at 24h intervals by RT-qPCR. 1 dpf ST-responsive expression of gbp4 **(B)** and gbp1 **(D)** was assessed in WT and KO groups. All qPCR data normalized to β-actin and expressed as fold change relative to 0h WT unstimulated control. *p < 0.05 versus unstimulated control WT; #*p* < 0.05 versus ST-stimulated WT.

The functional consequences of this disrupted signaling were evident in the expression of downstream effectors. The ISG gbp4 showed progressive constitutive expression in WT embryos (**Figure 6A**) and was powerfully upregulated over 200-fold by ST challenge. This induction was entirely abolished in natterin KO embryos (**Figure 6B**). Interestingly, gbp1 expression patterns differed; it was constitutively expressed but not induced by ST in WT embryos. However, gbp1 was significantly upregulated in KO embryos upon challenge (**Figures 6C, D**), implying a compensatory mechanism may be activated in the absence of the primary Gbp4 defense pathway.

**Figure 7:**
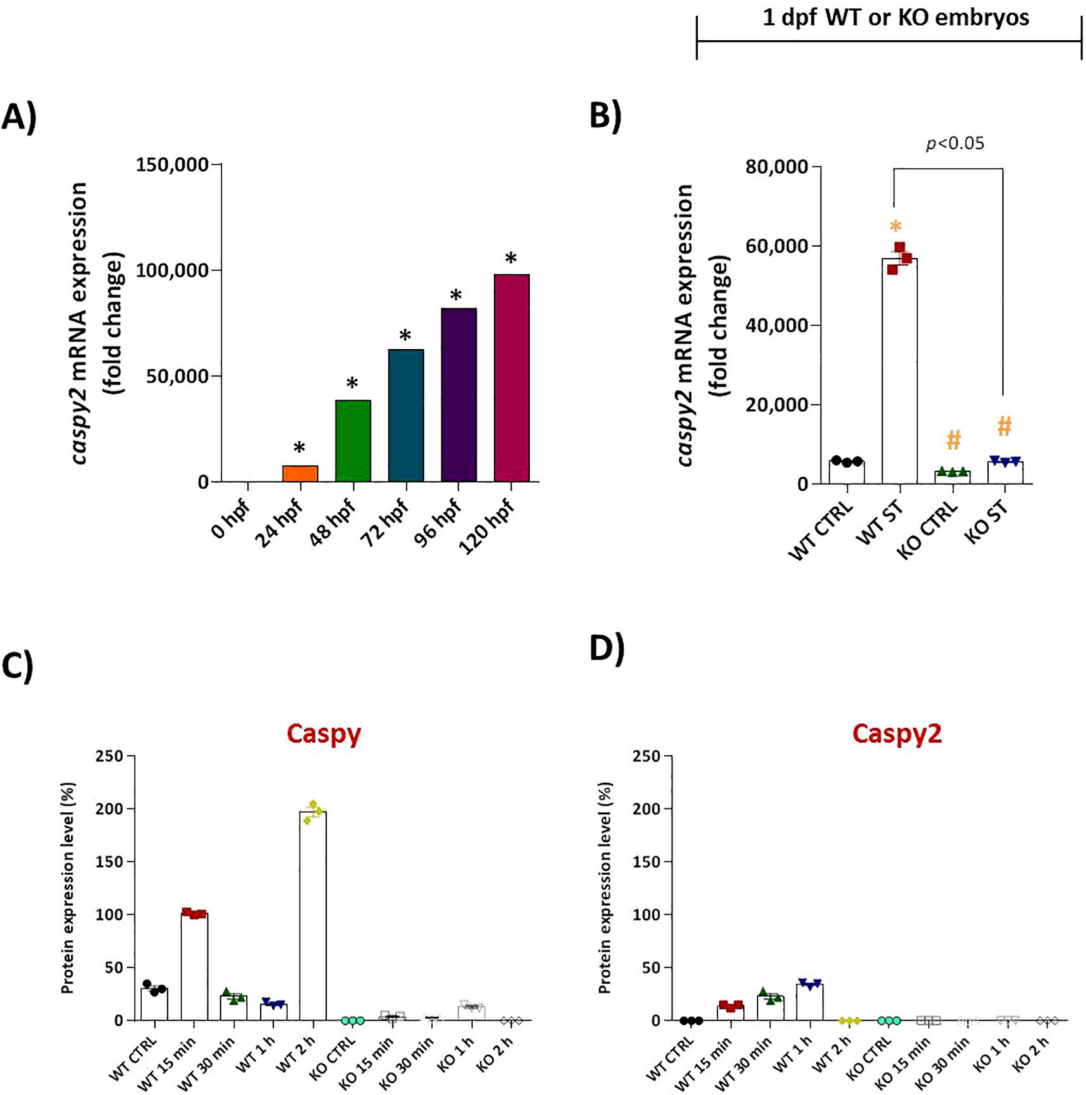
Natterin is essential for proteolytic activation of Caspy and Caspy2 during ST stimulation. **(A)** Developmental expression profile of caspy2 mRNA in unstimulated WT embryos (*n* = 100/group) from 24 to 120 hpf. **(B)** One day post-fertilization ST-induced caspy2 expression in WT versus natterin knockout (KO) embryos 2h post-stimulation. All qPCR data normalized to β-actin and expressed as fold change relative to 0h WT unstimulated control. Data represent mean ± SEM; *p < 0.05 versus unstimulated control WT, #*p* < 0.05 versus ST-stimulated WT. Western blot analysis of mature Caspy (**Supplementary Figure S3**) and Caspy2 (**Supplementary Figure S4**) expression in whole embryo lysates at indicated times post-ST stimulation (15 and 30 min, 1 and 2h). Data are normalized to the unstimulated control and presented as percentage values. Quantitative densitometry is shown in bar graphs **(C, D)**.

We then focused on the core inflammasome component, caspy2. Unstimulated WT embryos exhibited high constitutive caspy2 mRNA expression, which was further enhanced 10-fold by ST challenge. Once again, natterin KO embryos completely lacked detectable caspy2 transcripts, both basally and after stimulation (**Figures 7A, B**).

To confirm these transcriptional defects translated to a functional protein deficiency, we analyzed Caspy and Caspy2 protein maturation by Western blot. In ST-stimulated WT embryos, the mature, active forms of Caspy (25–28 kDa) and Caspy2 were clearly detected within 15 min, with strong expression sustained for up to 2h (**Supplementary Figures 3A**, **4A**). In dramatic contrast, ST-stimulated natterin KO embryos showed only faint, transient levels of mature Caspy and a complete absence of mature Caspy2 (mCaspy2) at all time points analyzed (**Figures 4B**, **5B**). Quantitative analysis of the blots confirmed this drastic reduction in protein expression (**Figures 7C, D**).

Collectively, these results demonstrate that Natterin is a non-redundant upstream regulator essential for the activation of the Gbp4/Caspy2 axis, governing both the transcriptional induction and functional proteolytic maturation required for non-canonical inflammasome activation in response to bacterial stimulation.

### 3.4 Natterin is essential for gasdermin-mediated pore formation

In zebrafish, the gasdermin isoforms *GSDME-a* and *GSDME-b* are central effectors of inflammatory cell death. *GSDME-b* is cleaved by the inflammatory caspase Caspy2—a key step in non-canonical inflammasome activation—and can also be processed by caspase-8a/b (Yang et al., 2018; Chen et al., 2021; Varela et al., 2019; Lozano-Gil et al., 2022). In contrast, *GSDME-a* is cleaved by caspase-3 or caspase-8a (Wang et al., 2020), linking it to both pyroptotic and apoptotic pathways (Fadeel and Orrenius, 2005). These distinct proteolytic activation mechanisms position gasdermins as critical executors of host defense against intracellular bacteria.

**Figure 8:**
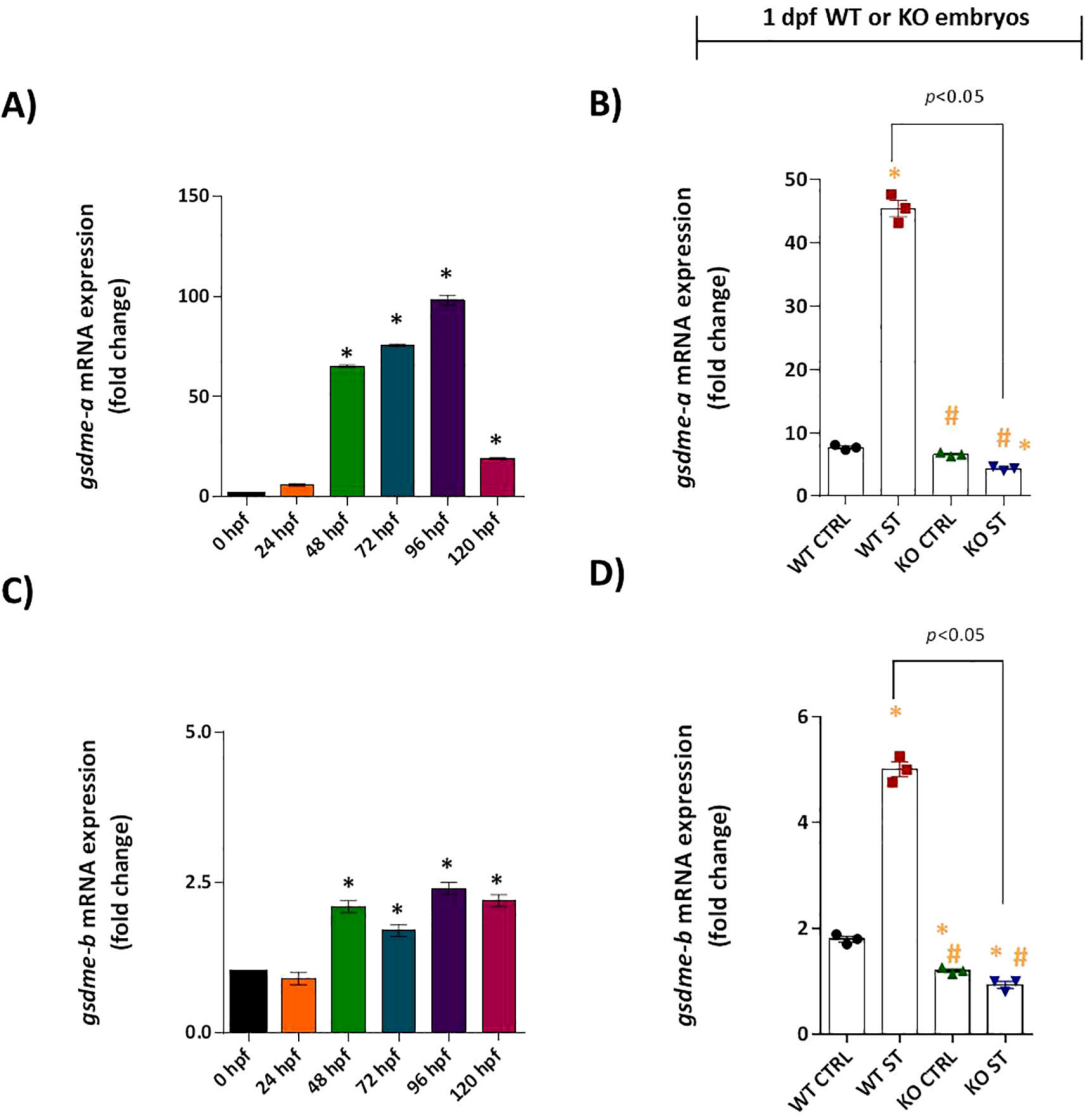
Role of Natterin in the gasdermin-dependent ST response. Constitutive *gsdme-a* **(A)** and *gsdme-b* **(C)** expression was analyzed in unstimulated WT embryos (*n* = 100/group) at 24-hour intervals by RT-qPCR. One day post-fertilization ST-stimulated WT and natterin KO embryos were assessed for *gsdme-a* **(B)** and *gsdme-b* **(D)** expression. All qPCR data normalized to β-actin and expressed as fold change relative to 0h WT unstimulated control. *p < 0.05 versus unstimulated control WT; #*p* < 0.05 versus ST-stimulated WT.

We investigated whether Natterin regulates this final step of the pyroptotic pathway by analyzing gasdermin expression. In wild-type (WT) embryos, *gsdme-a* mRNA was moderately expressed from 48 hpf, peaking at 96 hpf (**Figure 8A**). ST stimulation induced a strong 5.9-fold upregulation of *gsdme-a* (**Figure 8B**). Similarly, *gsdme-b* was expressed at low basal levels (**Figure 8C**) and was significantly upregulated 2.9-fold following challenge (**Figure 8D**). This coordinated induction suggests both isoforms play a role in the host response to ST.

Critically, this entire transcriptional response was entirely dependent on Natterin. The potent ST-induced upregulation of both *gsdme-a* and *gsdme-b* was completely abolished in natterin KO embryos (**Figures 8B, D**). To determine if the gasdermin pathway converged with apoptotic cascades, we assessed the expression of key apoptotic initiators. Neither caspase-3 nor caspase-8 mRNA was detectable in WT or KO embryos, with or without ST stimulation (**Supplementary Figures S6A–D**), effectively ruling out the involvement of the extrinsic apoptotic pathway in this context.

These findings position Natterin as a master regulator of the entire gasdermin-dependent effector response, governing the transcriptional induction of the pore-forming executioners *gsdme-a* and *gsdme-b* downstream of non-canonical inflammasome activation.

## 4 Discussion

Our study unveils a sophisticated immune network in zebrafish, demonstrating Natterin orchestrates host defense against ST by bridging type I interferon (IFN-I) signaling and inflammasome activation. This conclusion is supported by a combination of independent experimental approaches, including the use of wild-type embryos treated with IFN-I neutralizing antibody and CRISPR/Cas9 natterin (loc795232) knockout (KO) embryos, alongside integrated methodologies such as RT-qPCR, immunohistochemistry, Western blotting, and behavioral assays.

Our findings confirm that IFN-φ1 is indispensable for zebrafish survival during ST challenge, aligning with previous work demonstrating its critical role in clearing intracellular ST (Kim et al., 2016). Neutralization of IFN-φ led to a six-fold increase in mortality, severe developmental delays, and exacerbated hypoactivity. Immunohistochemistry and western blot data further confirmed that IFN-φ neutralization completely suppressed IL-1β maturation.

Surprisingly, Natterin functions as a critical upstream regulator essential for initiating the transcriptional response. We observed a downregulation of *irf3*, *irf7*, and *crfb1* mRNA in the absence of Natterin, which could impair type I interferon binding and signaling, thereby hindering the induction of interferon-stimulated genes (ISGs) such as gbp4. The concomitant downregulation of gbp4, caspy2 (along with impaired caspase maturation), and *gsdme-a/b* suggests a failure in cytosolic LPS sensing, caspase activation, and gasdermin-mediated pore formation—all essential components of an effective antibacterial response. This highlights an unexpected synergy between IFN-I and inflammasome pathways, contrasting with the traditional view of their antagonism (Guarda et al., 2011).

Our results align with emerging evidence in mammals where IFN-I enhances inflammasome activity (Aarreberg et al., 2018; Hong et al., 2020) and IL-1β reciprocally amplifies IFN-I production (Härtlova et al., 2015). Together our data reinforce the interdependence of these pathways in antibacterial immunity in zebrafish.

Mechanistically, we demonstrate that Natterin drives IFN-φ1 production through the *IRF3*/*IRF7* pathway, partially dependent on STING and independent of MyD88. While ST stimulation robustly upregulated *irf3* (39-fold) and *irf7* (ninefold) in wild-type (WT) embryos, natterin KO embryos failed to induce these transcription factors, indicating a critical role for Natterin in initiating IFN-I signaling. This suggests that Natterin may operate upstream of STING, which itself partially depends on Natterin for its induction, but not of MyD88, which remained unresponsive. These findings position Natterin as a key modulator of cytosolic immune sensing, potentially influencing the cGAS-STING or TRIF-*IRF3* pathways (Liu et al., 2015; Rathinam et al., 2012).

A major breakthrough of this study is the discovery that Natterin is required for the expression of *CRFB1* (the IFN-φ1 receptor), mature Caspy2 protein (a caspase-11 ortholog), and Gbp4—all essential components of the non-canonical inflammasome. While WT embryos exhibited strong upregulation of *crfb1* (16.5-fold), caspy2 (10-fold), and gbp4 (235-fold) following ST stimulation, KO embryos showed complete ablation of these transcripts and a corresponding absence of mature Caspy and Caspy2 proteins, explaining their immunocompromised phenotype. This aligns with mammalian studies where IFN-I primes cells for cytosolic LPS sensing by upregulating caspase-11 and Gbps (Meunier et al., 2014; Pilla et al., 2014). Notably, Gbp4—a zebrafish homolog of mammalian Gbp2—likely facilitates LPS release from bacterial vesicles, enabling Caspy2 activation (Tyrkalska et al., 2016; Yi, 2020). The absence of this axis in KO embryos underscores Natterin’s role in licensing non-canonical inflammasome assembly.

We further reveal that Natterin regulates gasdermin (GSDME)-dependent responses, as the induction of both *gsdme-a* and *gsdme-b* was abolished in KO embryos. Given that *GSDME-b* is cleaved by Caspy2 (Yang et al., 2018), its loss in KO embryos suggests impaired pyroptotic cell death due to the lack of functional Caspy2. However, the absence of caspase-3 and caspase-8 activation implies that extrinsic apoptosis is not engaged, reinforcing GSDME-mediated pyroptosis as the dominant cell death mechanism. Beyond lytic cell death, GSDME pores may facilitate IL-1β release and K^+^ efflux (Evavold et al., 2018; Rühl and Broz, 2022), potentially amplifying NLRP3 inflammasome activation—a mechanism that warrants further investigation.

Interestingly, KO embryos exhibited upregulation of gbp1 in response to ST, suggesting a compensatory response to gbp4 deficiency. The lack of restored immunity in KO embryos likely indicates that Gbp1 does not functionally overlap with Gbp4 in inflammasome activation. Additionally, the absence of crfb2 (an IFN-φ3 ligand) highlights the specificity of the IFN-φ1/*CRFB1* axis in zebrafish antibacterial defense.

The striking parallels between the IFN-φ-neutralized and natterin-knockout phenotypes highlight the non-redundant role of Natterin bridging interferon and inflammasome pathways (**Figure 9**). This work not only advances our understanding of innate immunity in teleosts but also provides evolutionary insights into the conserved crosstalk between cytosolic surveillance and inflammatory cell death in vertebrates. Future studies should focus on identifying functional analogs of Natterin in mammalian systems and elucidating whether they similarly govern the IFN-inflammasome axis.

Given the clinical relevance of this pathway in septic shock or chronic inflammatory autoimmune diseases, understanding such a master regulator could have significant translational implications. For instance, modulating a Natterin-like pathway may offer a novel strategy to fine-tune excessive inflammation or boost antibacterial activity. This IFN-inflammasome axis, orchestrated by Natterin, represents a novel immune checkpoint with potential therapeutic implications across species.

## Data Availability

The sequence and information for the gene natterin (accession number 795232) are available through the NCBI Gene repository at the following URL: https://www.ncbi.nlm.nih.gov/gene/?term=loc795232. All other raw data supporting the conclusions of this article, are included in the article and its Supplementary Material or are available from the corresponding author upon reasonable request.

## Supplementary material

Supplementary Figure 1
**|** Original Western blot showing Caspy2 expression in lysates from anti-IFNβ-neutralized embryos. Independent groups of 1 dpf embryos (*n* = 80/group) were neutralized by immersion in fresh 0.5× E2 medium containing a monoclonal mouse IgG2a anti-human IFN-β neutralizing antibody for 30 min and then exposed or not to *Salmonella typhimurium* (ST) for 2h. Lysates from the different groups containing 5 µg of protein were subjected to electrophoresis and identification of Caspy2 **(A)** using a rabbit polyclonal IgG primary antibody raised against amino acids 301–350 of caspase-11 of mouse origin (p-10 M-50), followed by a rabbit HRP-labeled anti-rabbit IgG TrueBlot secondary antibody. As an internal control, β-actin **(B)** was identified using a mouse monoclonal IgG1κ anti-mouse pan-actin clone C4 (43 kDa), followed by an anti-mouse IgG HRP secondary antibody. Revelation was performed by adding SuperSignal West Femto Maximum Sensitivity Substrate and imaging with an Amersham Imager 680 photodocumenter, with an exposure time of 1 s.

Supplementary Figure 2 Original Western blot showing Natterin expression in WT embryos stimulated with ST and subjected to various treatments compared to KO embryos. Independent groups of 1 dpf embryos (*n* = 80/group) were pretreated by immersion in fresh 0.5× E2 medium for 1h with MCC950 or Pam3CSK4 and then exposed or not to ST for 2h. WT embryos were also exposed or not to ST for 2h. loc795232 KO embryos were stimulated with ST for 2h. Lysates from the different groups containing 5 µg of protein were subjected to electrophoresis and identification of Natterin **(A)** using a rabbit anti-serum against natterin purified from Thalassophryne nattereri venom (dimeric form around 62 kDa), followed by an anti-rabbit IgG HRP TrueBlot secondary antibody. As an internal control, β-actin **(B)** was identified using a mouse monoclonal IgG1κ anti-mouse pan-actin clone C4 (43 kDa), followed by an anti-mouse IgG HRP secondary antibody. Revelation was performed by adding SuperSignal West Femto Maximum Sensitivity Substrate and imaging with an Amersham Imager 680 photodocumenter, with an exposure time of 1 s.

Supplementary Figure 3
**|** Original Western blot showing Caspy expression in WT and KO embryos. WT or loc795232 KO embryos were stimulated for different times (15 min, 30 min, 1h, or 2h) with Staphylococcus aureus or *Salmonella typhimurium* at 10^6 cells/ml and used for identification of Caspy. Lysates from the different WT **(A)** or KO **(B)** groups containing 5 µg of protein were subjected to electrophoresis and identification of Caspy using a rabbit polyclonal IgG anti-mouse caspase-1 primary antibody (p-10, M-20), followed by a rabbit HRP-labeled anti-rabbit IgG TrueBlot secondary antibody. Revelation was performed by adding SuperSignal West Femto Maximum Sensitivity Substrate and imaging with an Amersham Imager 680 photodocumenter, with an exposure time of 1 s. Blots shown are representative of three independent experiments.

Supplementary Figure 4
**|** Original Western blot showing Caspy2 expression in WT and KO embryos. WT or loc795232 KO embryos were stimulated for different times (15 min, 30 min, 1h, or 2h) with Staphylococcus aureus or *Salmonella typhimurium* at 10^6 cells/ml and used for identification of Caspy2. Lysates from the different WT **(A)** or KO **(B)** groups containing 5 µg of protein were subjected to electrophoresis and identification of Caspy2 using a rabbit polyclonal IgG primary antibody raised against amino acids 301–350 of caspase-11 of mouse origin (p-10, M-50), followed by a rabbit HRP-labeled anti-rabbit IgG TrueBlot secondary antibody. Revelation was performed by adding SuperSignal West Femto Maximum Sensitivity Substrate and imaging with an Amersham Imager 680 photodocumenter, with an exposure time of 1 s. Blots shown are representative of three independent experiments.

Supplementary Figure 5
**|** MyD88 and CRFB2 show no response to ST challenge. **(A)** Developmental expression profile of myd88 mRNA in unstimulated WT embryos (*n* = 100/group) from 24 to 120 hpf. **(B)** Myd88 expression in 1 dpf WT and natterin KO embryos 2h post-ST stimulation. **(C)** Baseline expression of crfb2 in unstimulated WT embryos. **(D)** crfb2 expression following ST stimulation in 1 dpf WT and KO embryos. All qPCR data normalized to β-actin and expressed as fold-change relative to 0 h WT controls. Data represent mean ± SEM of three biological replicates.

Supplementary Figure 6 Apoptotic caspases remain repressed during ST stimulation. **(A)** Developmental trajectory of casp-3 expression in unstimulated WT embryos (*n* = 100/group). **(B)** casp-3 expression in 1 dpf WT and KO embryos after ST stimulation. **(C)** Constitutive expression profile of casp-8 in control embryos. **(D)** 1 dpf ST-responsive casp-8 expression in WT versus KO embryos. All qPCR data normalized to β-actin and expressed as fold change relative to 0h WT unstimulated control. Data represent mean ± SEM from three independent experiments.
