# Supplementary figures and images for: Natterin bridges IFN-φ1 and non-canonical inflammasome pathways via CRFB1/Gbp4 to license Caspy2-mediated antibacterial immunity
Source: Front Cell Infect Microbiol. 2025 Oct 27;15:1686758. doi: 10.3389/fcimb.2025.1686758 (PMC12597722; doi:10.3389/fcimb.2025.1686758)

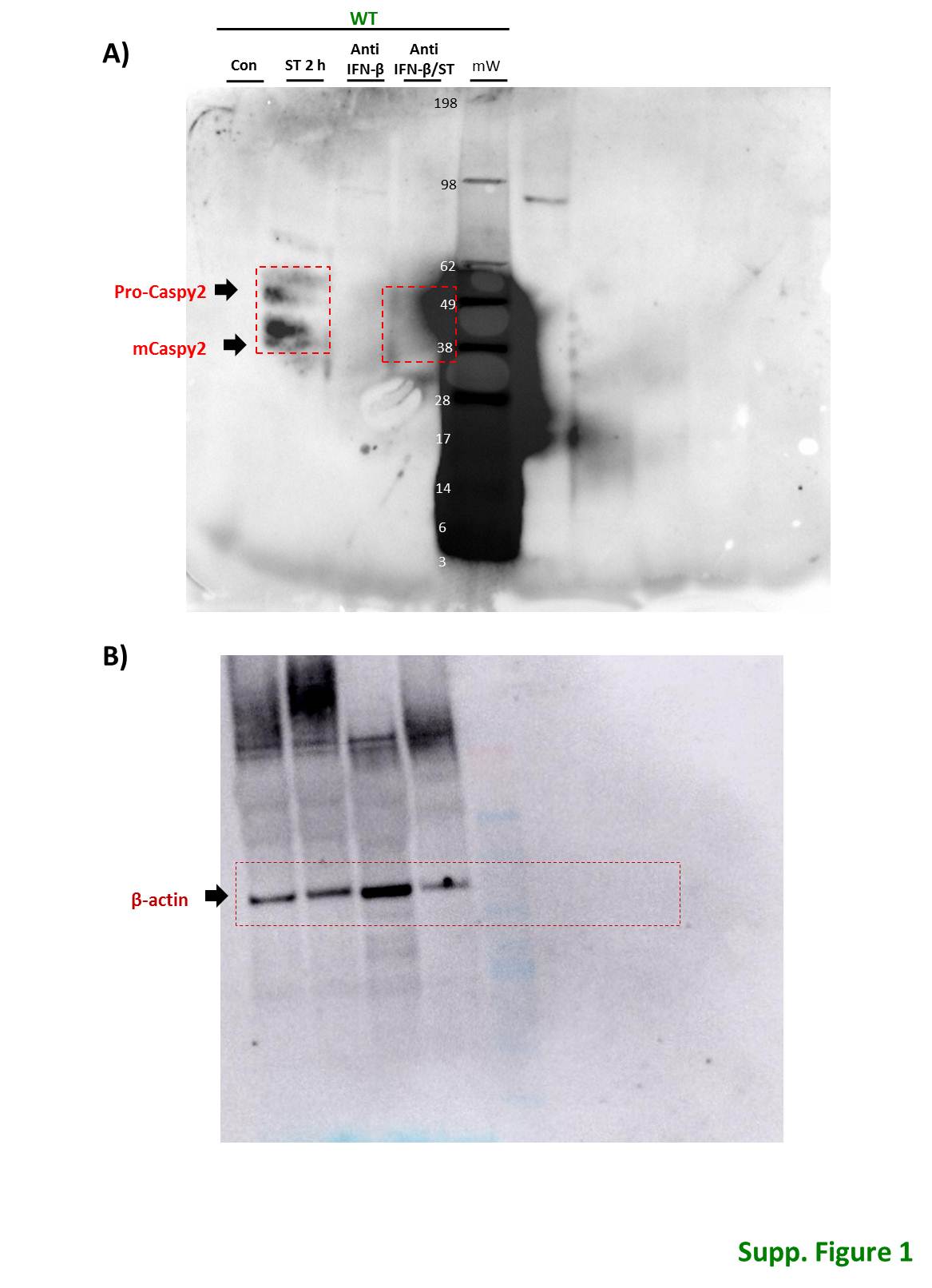

Supplement: Supplementary Figure 1 — | Original Western blot showing Caspy2 expression in lysates from anti-IFNβ-neutralized embryos. Independent groups of 1 dpf embryos (n = 80/group) were neutralized by immersion in fresh 0.5× E2 medium containing a monoclonal mouse IgG2a anti-human IFN-β neutralizing antibody for 30 min and then exposed or not to Salmonella typhimurium (ST) for 2h. Lysates from the different groups containing 5 µg of protein were subjected to electrophoresis and identification of Caspy2 (A) using a rabbit polyclonal IgG primary antibody raised against amino acids 301–350 of caspase-11 of mouse origin (p-10 M-50), followed by a rabbit HRP-labeled anti-rabbit IgG TrueBlot secondary antibody. As an internal control, β-actin (B) was identified using a mouse monoclonal IgG1κ anti-mouse pan-actin clone C4 (43 kDa), followed by an anti-mouse IgG HRP secondary antibody. Revelation was performed by adding SuperSignal West Femto Maximum Sensitivity Substrate and imaging with an Amersham Imager 680 photodocumenter, with an exposure time of 1 s. [file Image1.jpeg]

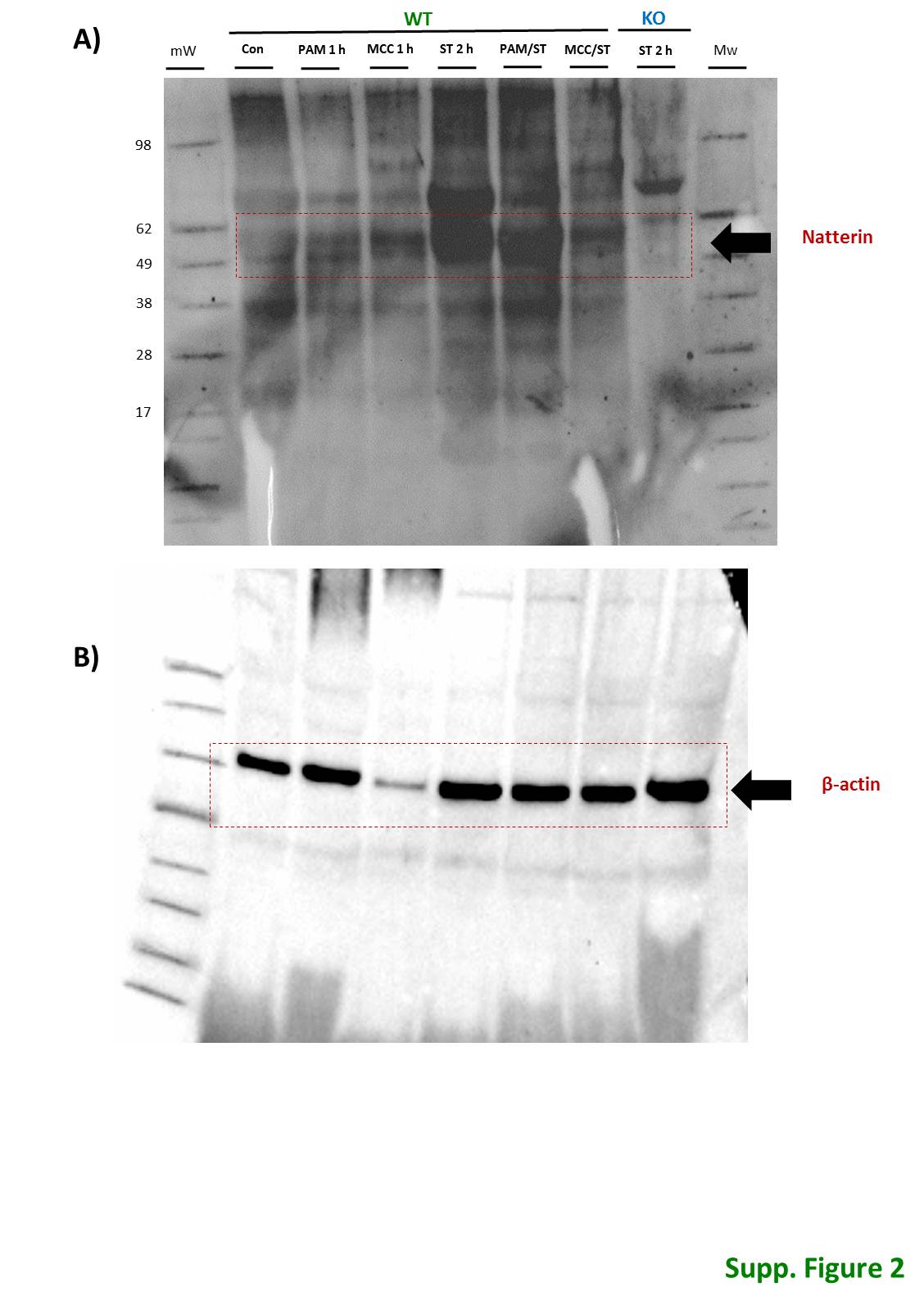

Supplement: Supplementary Figure 2 — Original Western blot showing Natterin expression in WT embryos stimulated with ST and subjected to various treatments compared to KO embryos. Independent groups of 1 dpf embryos (n = 80/group) were pretreated by immersion in fresh 0.5× E2 medium for 1h with MCC950 or Pam3CSK4 and then exposed or not to ST for 2h. WT embryos were also exposed or not to ST for 2h. loc795232 KO embryos were stimulated with ST for 2h. Lysates from the different groups containing 5 µg of protein were subjected to electrophoresis and identification of Natterin (A) using a rabbit anti-serum against natterin purified from Thalassophryne nattereri venom (dimeric form around 62 kDa), followed by an anti-rabbit IgG HRP TrueBlot secondary antibody. As an internal control, β-actin (B) was identified using a mouse monoclonal IgG1κ anti-mouse pan-actin clone C4 (43 kDa), followed by an anti-mouse IgG HRP secondary antibody. Revelation was performed by adding SuperSignal West Femto Maximum Sensitivity Substrate and imaging with an Amersham Imager 680 photodocumenter, with an exposure time of 1 s. [file Image2.jpeg]

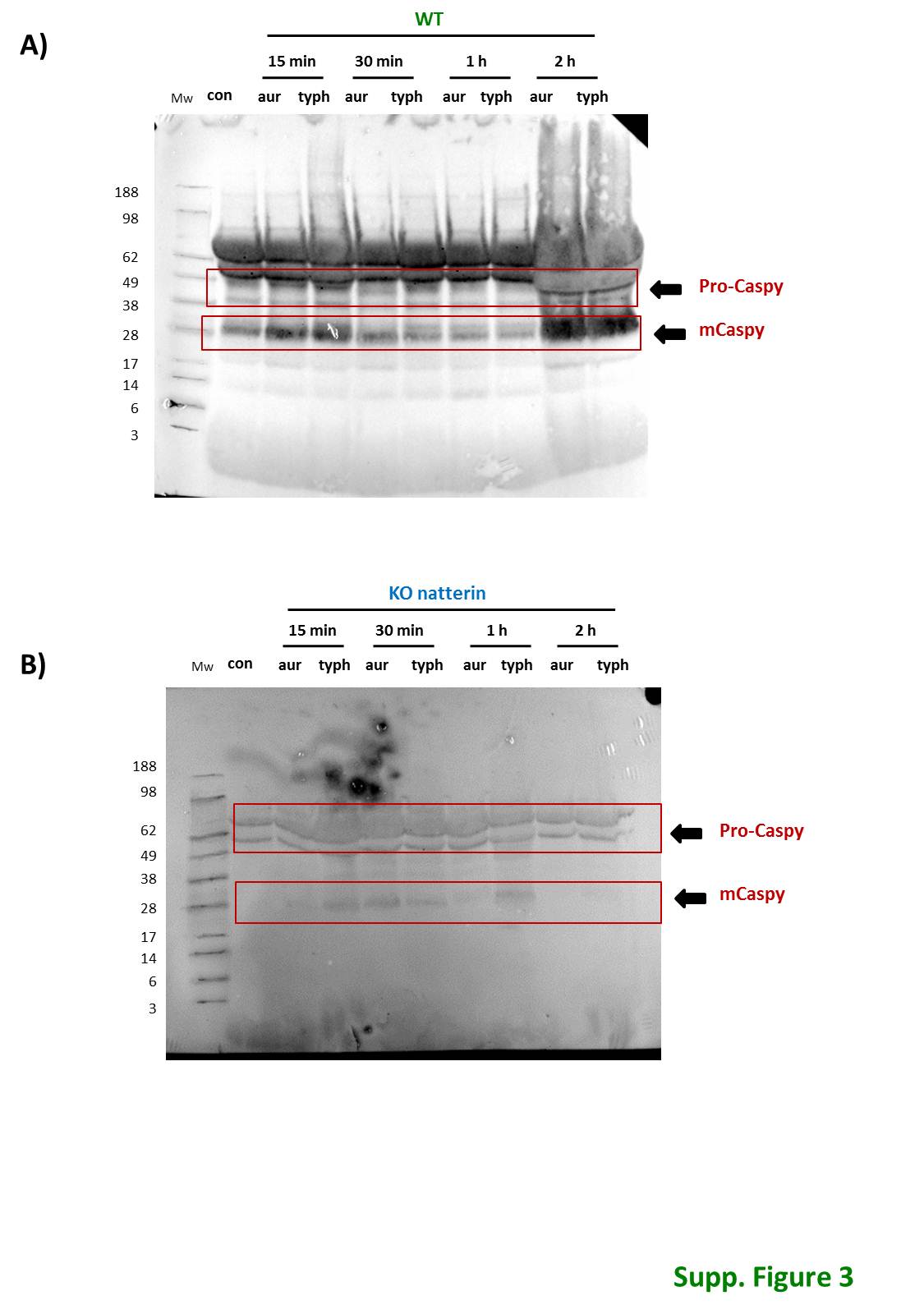

Supplement: Supplementary Figure 3 — | Original Western blot showing Caspy expression in WT and KO embryos. WT or loc795232 KO embryos were stimulated for different times (15 min, 30 min, 1h, or 2h) with Staphylococcus aureus or Salmonella typhimurium at 10^6 cells/ml and used for identification of Caspy. Lysates from the different WT (A) or KO (B) groups containing 5 µg of protein were subjected to electrophoresis and identification of Caspy using a rabbit polyclonal IgG anti-mouse caspase-1 primary antibody (p-10, M-20), followed by a rabbit HRP-labeled anti-rabbit IgG TrueBlot secondary antibody. Revelation was performed by adding SuperSignal West Femto Maximum Sensitivity Substrate and imaging with an Amersham Imager 680 photodocumenter, with an exposure time of 1 s. Blots shown are representative of three independent experiments. [file Image3.jpeg]

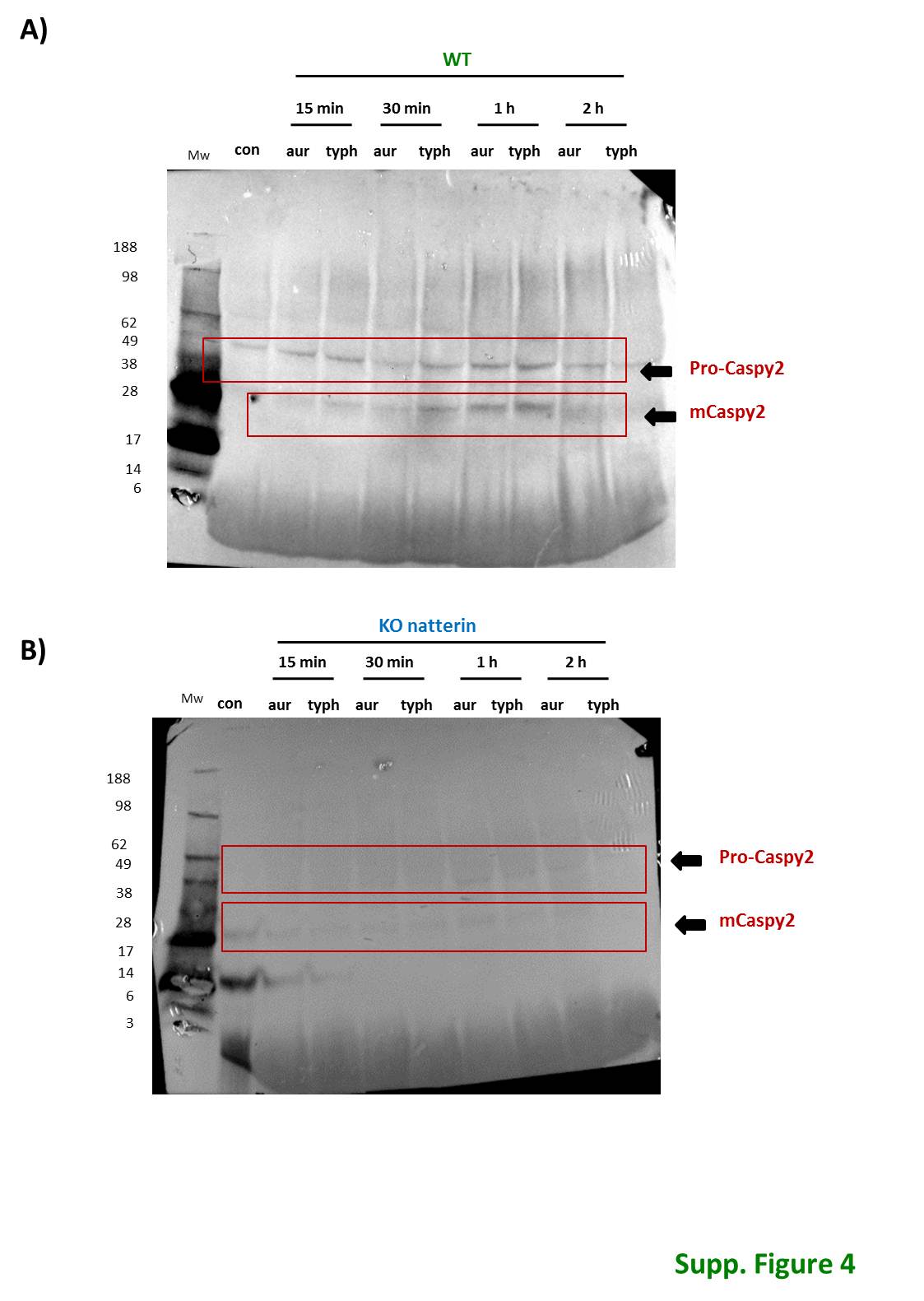

Supplement: Supplementary Figure 4 — | Original Western blot showing Caspy2 expression in WT and KO embryos. WT or loc795232 KO embryos were stimulated for different times (15 min, 30 min, 1h, or 2h) with Staphylococcus aureus or Salmonella typhimurium at 10^6 cells/ml and used for identification of Caspy2. Lysates from the different WT (A) or KO (B) groups containing 5 µg of protein were subjected to electrophoresis and identification of Caspy2 using a rabbit polyclonal IgG primary antibody raised against amino acids 301–350 of caspase-11 of mouse origin (p-10, M-50), followed by a rabbit HRP-labeled anti-rabbit IgG TrueBlot secondary antibody. Revelation was performed by adding SuperSignal West Femto Maximum Sensitivity Substrate and imaging with an Amersham Imager 680 photodocumenter, with an exposure time of 1 s. Blots shown are representative of three independent experiments. [file Image4.jpeg]

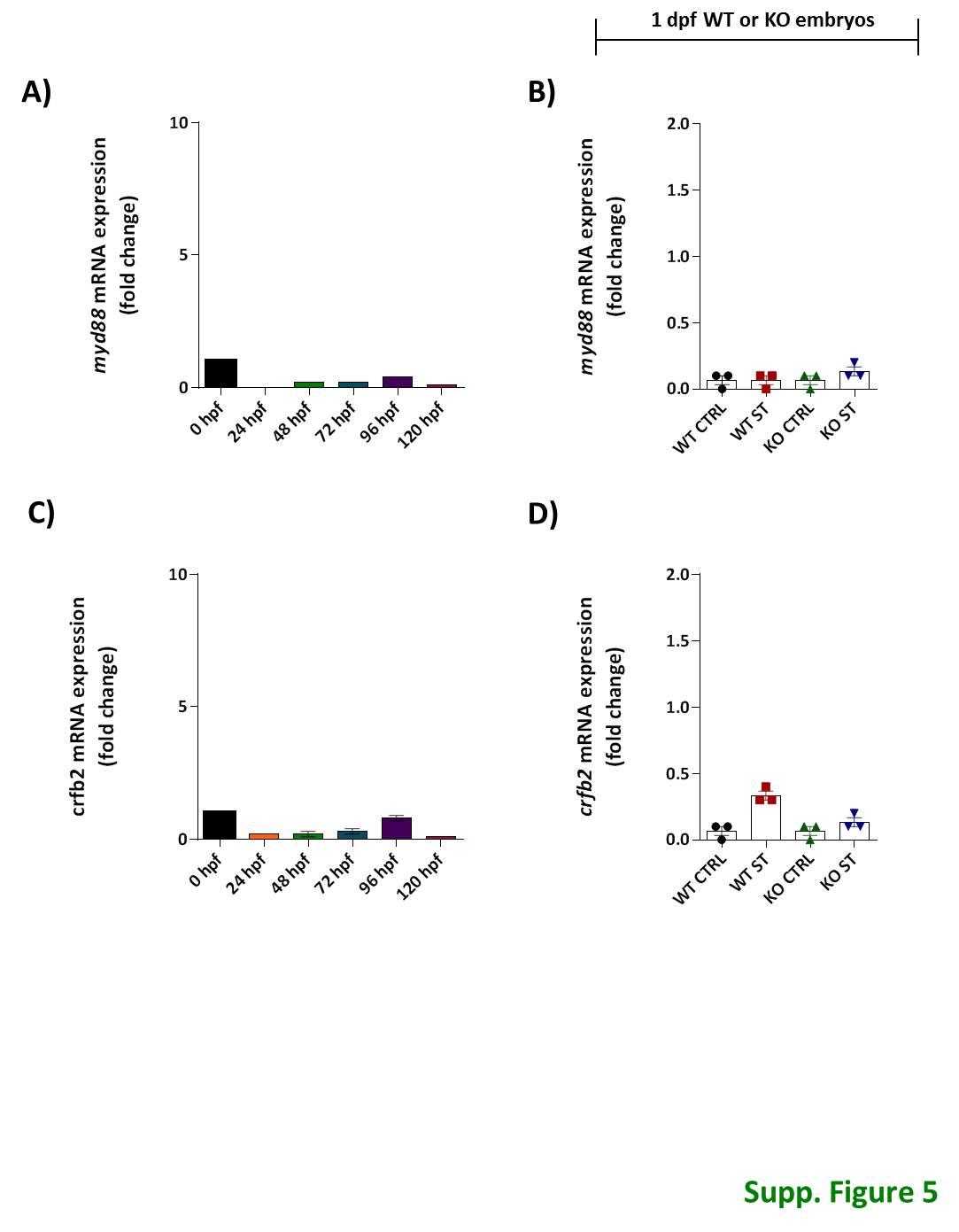

Supplement: Supplementary Figure 5 — | MyD88 and CRFB2 show no response to ST challenge. (A) Developmental expression profile of myd88 mRNA in unstimulated WT embryos (n = 100/group) from 24 to 120 hpf. (B) Myd88 expression in 1 dpf WT and natterin KO embryos 2h post-ST stimulation. (C) Baseline expression of crfb2 in unstimulated WT embryos. (D) crfb2 expression following ST stimulation in 1 dpf WT and KO embryos. All qPCR data normalized to β-actin and expressed as fold-change relative to 0 h WT controls. Data represent mean ± SEM of three biological replicates. [file Image5.jpeg]

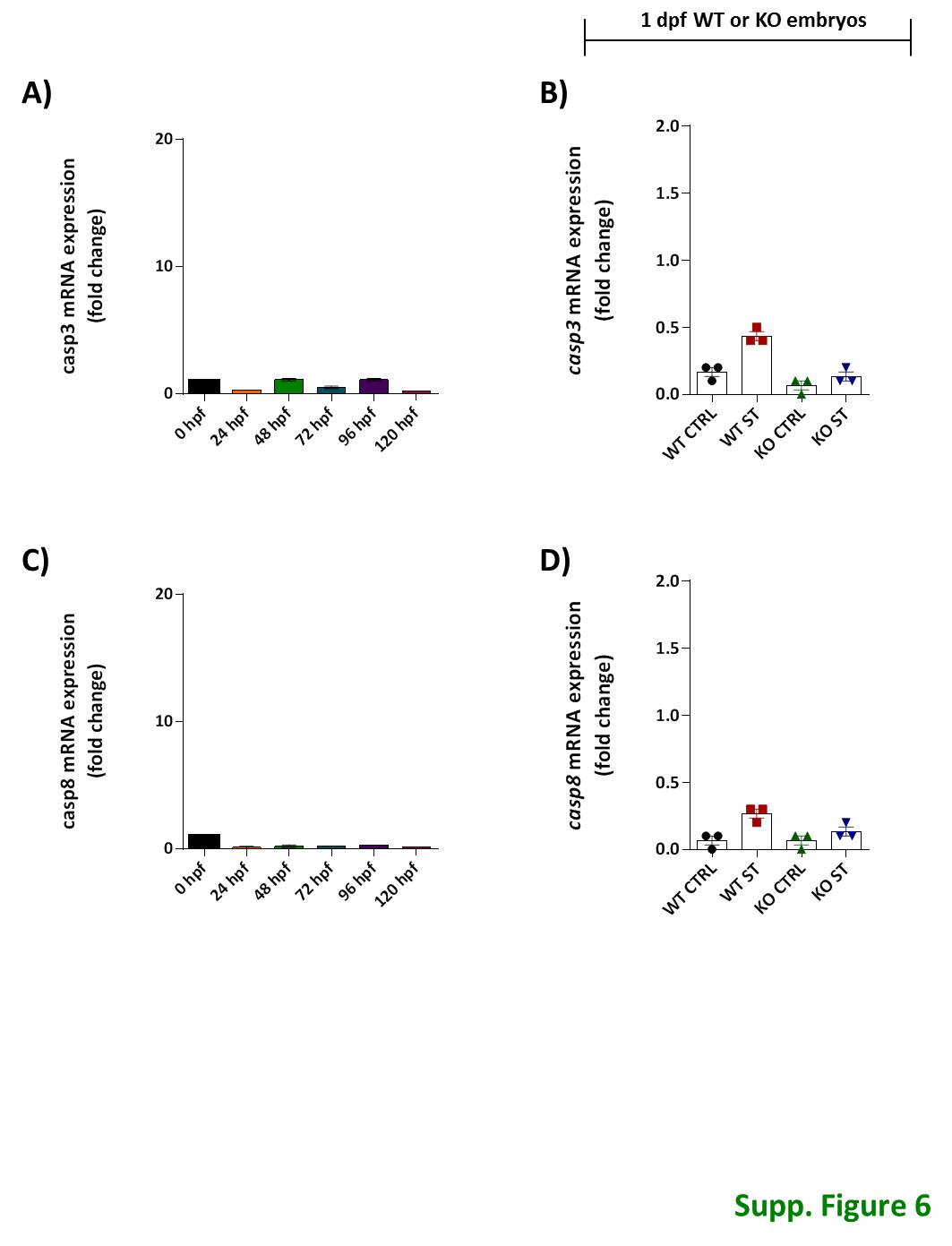

Supplement: Supplementary Figure 6 — Apoptotic caspases remain repressed during ST stimulation. (A) Developmental trajectory of casp-3 expression in unstimulated WT embryos (n = 100/group). (B) casp-3 expression in 1 dpf WT and KO embryos after ST stimulation. (C) Constitutive expression profile of casp-8 in control embryos. (D) 1 dpf ST-responsive casp-8 expression in WT versus KO embryos. All qPCR data normalized to β-actin and expressed as fold change relative to 0h WT unstimulated control. Data represent mean ± SEM from three independent experiments. [file Image6.jpeg]
